# Depot and sex‐specific implications for adipose tissue expandability and functional traits in adulthood of late prenatal and early postnatal malnutrition in a precocial sheep model

**DOI:** 10.14814/phy2.14600

**Published:** 2020-10-10

**Authors:** Sharmila Ahmad, Lise Kirstine Lyngman, Morteza Mansouryar, Rajan Dhakal, Jørgen Steen Agerholm, Prabhat Khanal, Mette Olaf Nielsen

**Affiliations:** ^1^ Nutrition Research Unit Department of Animal Science Aarhus University Tjele Denmark; ^2^ Section of Production, Nutrition and Health Department of Veterinary and Animal Sciences University of Copenhagen Frederiksberg C Denmark; ^3^ Section for Reproduction and Obstetrics Department of Veterinary Clinical Sciences University of Copenhagen Taastrup Denmark; ^4^ Faculty of Biosciences and Aquaculture Division for Animal science, Production and Welfare Nord University Steinkjer Norway

**Keywords:** early life malnutrition, epicardial adipose tissue, gene expression, mesenteric adipose tissue, perirenal adipose tissue, subcutaneous adipose tissue

## Abstract

The aim was to investigate long‐term, tissue and sex‐specific impacts of pre and postnatal malnutrition on expandability and functional traits of different adipose tissues. Twin‐pregnant ewes were fed NORM (~requirements), LOW (50% of NORM) or HIGH (150%/110% of energy/protein) diets the last 6 weeks prepartum (term ~147‐days). Lambs received moderate, low‐fat (CONV) or high‐carbohydrate‐high‐fat (HCHF) diets from 3 days until 6 months of age, and thereafter CONV diet. At 2½ years of age (adulthood), histomorphometric and gene expression patterns were characterized in subcutaneous (SUB), perirenal (PER), mesenteric (MES), and epicardial (EPI) adipose tissues. SUB had sex‐specific (♂<♀) upper‐limits for adipocyte size and cell‐number indices, irrespective of early life nutrition. PER mass and contents of adipocytes were highest in females and HIGH♂, whereas adipocyte cross‐sectional area was lowest in LOW♂. Pre/postnatal nutrition affected gene expression sex‐specifically in SUB + PER, but unrelated to morphological changes. In PER, LOW/LOW♂ were specific targets of gene expression changes. EPI was affected by postnatal nutrition, and HCHF sheep had enlarged adipocytes and upregulated expressions for adipogenic and lipogenic genes. Conclusion: upper‐limits for SUB expandability were markedly lower in males. Major targets for prenatal malnutrition were PER and males. LOW♂ had the lowest PER expandability, whereas HIGH♂ had an adaptive advantage due to increased hypertrophic ability equivalent to females. Fixed expandability in SUB meant PER became a determining factor for MES and ectopic fat deposition, rendering LOW♂ particularly predisposed for obesity‐associated metabolic risks. EPI, in contrast to other tissues, was targeted particularly by early postnatal obesity, resulting in adipocyte hypertrophy in adulthood.

## Graphical abstract

Iron hematoxylin, Adipose Tissue App (Visiopharm^®^ software with application (APP) name; Iron Haematoxylin, Adipose Tissue (APP ID 10113), version 1.0). (A) Adipose cells classification based on Form Factor (classes range from a Form Factor value of 0 (red; straight line) to 1 (dark blue; perfect circle), as indicated by the color bar). (B) Adipose cells classification based on cross‐sectional area (CSA; classes range from a CSA of 60 um^2^ (red) to 36,000 μm^2^ (dark blue), as indicated by the color bar). (C) Membrane and cell area detected by the APP “02 Quantify Fat Cell Membranes”. (D) Example of stained adipose tissue upon which the App is applied.

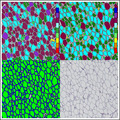

## 1 BACKGROUND

Pre and postnatal malnutrition have distinct impacts on key metabolic organs (Guan et al., 2005; Long et al., 2015) and adverse consequences for health during the entire lifespan. White adipose tissue formation and differentiation take place during fetal development and continues into the early postnatal period (Symonds et al., 2003). Previous studies have demonstrated that nutritional perturbations at different stages of gestation can alter adipocyte morphology and key genes involved in the regulation of adipose tissue development, leading to postnatal alterations in the functional properties and accumulation of body fat (Long et al., 2015; Muhlhausler et al., 2008). We have previously shown that both maternal over‐ and undernutrition (HIGH and LOW, respectively) during late gestation alter fat deposition patterns as well as adipose gene expression patterns in adolescent sheep and rats (Khanal et al., 2014, 2020; Kjaergaard et al., 2017; Nielsen et al., 2013). This could be ascribed to suppressed expandability of subcutaneous adipose tissue (SUB), resulting in a predisposition for visceral adiposity upon early postnatal development of obesity.

SUB is believed to play a key role in the partitioning of fat deposition (Moreno‐Indias & Tinahones, 2015), and it counteracts fat deposition elsewhere by acting as an energy “sink” in periods of excess energy intake (Tchkonia et al., 2013). However, the storage capacity of a single adipocyte appears to be finite (Moreno‐Indias & Tinahones, 2015). According to the adipose tissue expandability hypothesis (Mittendorfer, 2011), once the limit for SUB expandability is exceeded, there is therefore an increased risk of redirection of lipid deposition toward other adipose tissues and/or nonlipocyte cell types. As reviewed by Tan and Pidal‐Pulg (Tan & Vidal‐Puig, 2008), the risk of metabolic disturbances associated with obesity is not so much linked to the amount of fat deposited in the body per se, but primarily to the expandability of adipose tissues and hence capacity for uptake and storage of excess nutrients. Furthermore, a balance between adipogenesis, lipogenesis and lipolysis controlling adipocyte hyperplasia and hypertrophy at the molecular and cellular levels plays an important role in the development of adipose‐related metabolic diseases such as type II diabetes, insulin resistance, and hyperlipidemia (Choe et al., 2016; Dubois et al., 2006). Adipocyte hypertrophy, but not hyperplasia, is most often associated with the occurrence of metabolic disturbances by inducing the expression of pro‐inflammatory cytokines such as *interleukins* (*IL*s)‐*6*/*8* and *monocyte chemoattractant protein‐1* (*MCP1*), hence predisposing for adipose tissue inflammation (reviewed in Choe et al. 2016).

Sex is known to have a major impact on body fat distribution, and males are more susceptible for visceral adiposity and obesity‐related diseases than females (Bloor et al., 2013), although the underlying mechanisms for these gender differences are not well understood. It has been shown that sex‐specific differences in expression of molecular markers of fat tissue differentiation and/or function appear to emerge already *in utero* (Tchoukalova et al., 2014; Wallace et al., 2015), and hence the sex‐specific phenotypic manifestation of traits could potentially be sensitive to nutritional insults during gestation. A study in baboons (*Papio* sp.), demonstrated that maternal suboptimal nutrition during mid‐gestation suppressed the growth of male, but not female, offspring and led to adipocyte hypertrophy accompanied by increased markers of white and brown‐type adipogenesis in omental fat (Tchoukalova et al., 2014). Another study in sheep also showed that young males are at greater risk than females to the onset of comorbidities associated with juvenile‐onset obesity, as they had a higher storage capacity of lipids within perirenal‐abdominal adipocytes and exhibited raised insulin levels and upregulation of inflammatory markers (Bloor et al., 2013). Similarly, in sheep, a low birth weight combined with a high fractional growth rate in early postnatal life was associated with an increased expression of lipogenic genes in males only (Muhlhausler et al., 2008).

It is noteworthy that the majority of rodent and human studies addressing long‐term implications of fetal nutrition have involved male individuals only, and the female “side of the story” is much less elucidated. In addition, the vast majority of experimental animal studies pertaining to fetal programming have been conducted in altricial species, such as rodents, where the offspring do not undergo an intrauterine development equivalent to third‐trimester development in humans.

In this study, we aimed to test the hypotheses that mismatching combinations of late gestation and early postnatal malnutrition have adverse implications for adipose expandability and functional traits in adulthood, which are differentially manifested in SUB, mesenteric (MES), perirenal (PER) and epicardial (EPI) adipose tissues, and in a sex‐specific way. To test this hypothesis, we used a well‐documented precocial animal model, the Copenhagen sheep model, for fetal programming (Khanal et al., 2014, 2015, 2016). The sheep had been exposed to LOW, adequate (NORM) or HIGH levels of nutrition during the last trimester of fetal development, followed by a restricted CONV or obesogenic, high‐fat HCHF diet from 3‐days until 6 months of age (right after puberty), and finally, a CONV diet fed ad libitum during the next 2 years. Adipose tissues were sampled at autopsy from the adult sheep when they reached the age of 2½ years.

## 2 MATERIAL AND METHODS

### 2.1 Experimental design, animals and diets

**FIGURE 1:**
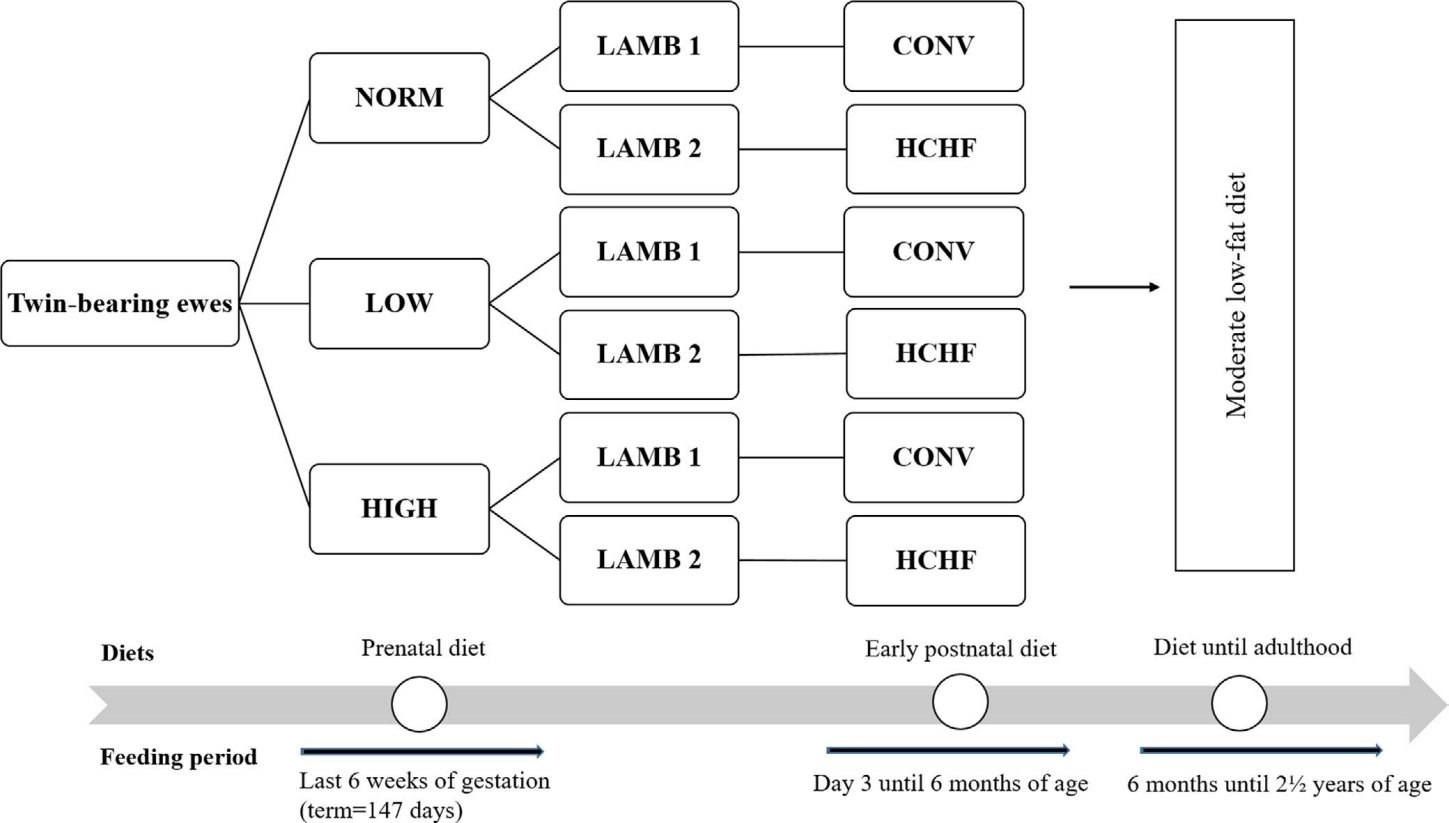
The flow chart of the experimental design and treatment groups. In short, a 3 (prenatal nutrition) x 2 (early postnatal nutrition) factorial design experiment was conducted, where 36 twin pregnant multiparous ewes were allocated to one of the following diets during the last 6 weeks of gestation (term ~ 147 days): NORM (fulfilling 100% of daily energy and protein requirements, LOW (providing 50% of NORM), or HIGH (providing 150% of daily energy and 110% of daily protein requirements). From 3 days after birth until 6 months of age (i.e. after puberty), one twin lamb from each dam was fed a low‐fat, moderate CONV diet (hay supplemented during the first 8 weeks of life with milk replacer; amounts were adjusted to ensure moderate growth rates of appr. 225 g/d). The other twin lamb was fed an obesogenic, high‐carbohydrate‐high‐fat HCHF diet (37% fat dairy cream with milk replacer in a 1:1 ratio (max. 2½ l/d) supplemented with rolled maize (max. 2 kg/d) and barley straw). Subgroups of lambs from each of the 6 treatment groups exited the experiment at 6 months of age. Remaining animals (*N* = 36) continued in the part of the experiment reported here to be studied as adult sheep, and they were from 6 months until 2½ years of age (adulthood) fed the same low‐fat hay‐based diet (hay ad libitum*;* supplemented until approx. 1 year of age with barley). The total number of animals in each of the six treatment groups were: NORM:CONV (*N* = 6); NORM:HCHF (*N* = 4); HIGH:CONV (*N* = 6); HIGH:HCHF (*N* = 6); LOW:CONV (*N* = 8) and; LOW:HCHF (*N* = 6)

The Copenhagen sheep model, experimental design, and dietary interventions have been described in detail previously (Khanal et al., 2014, 2016). All the experimental animal handling procedures were approved by the Danish National Committee on Animal Experimentation. In short, a 3 (prenatal nutrition) × 2 (early postnatal nutrition) factorial design experiment was conducted, where 36 twin pregnant multiparous ewes were allocated to one of the following diets during the last 6 weeks of gestation (term ~147 days): NORM (fulfilling 100% of daily energy and protein requirements, LOW (providing 50% of NORM), or HIGH (providing 150% of daily energy and 110% of daily protein requirements; Figure 1). From 3 days after birth until 6 months of age (i.e., after puberty), one twin lamb from each dam was fed a low‐fat, moderate CONV diet (hay supplemented during the first 8 weeks of life with milk replacer; amounts were adjusted to ensure moderate growth rates of appr. 225 g/d). The other twin lamb was fed an obesogenic, high‐carbohydrate‐high‐fat HCHF diet (37% fat dairy cream with milk replacer in a 1:1 ratio (max. 2½ l/d) supplemented with rolled maize (max. 2 kg/d) and barley straw). Subgroups of lambs from each of the 6 treatment groups exited the experiment at 6 months of age. Remaining animals (*N* = 36) continued in the part of the experiment reported here to be studied as adult sheep, and they were from 6 months until 2½ years of age (adulthood) fed the same low‐fat hay‐based diet (hay ad libitum*;* supplemented until approx. 1 year of age with barley). The total number of animals in each of the six treatment groups was NORM‐CONV (*N* = 6); NORM‐HCHF (*N* = 4); HIGH‐CONV (*N* = 6); HIGH‐HCHF (*N* = 6); LOW‐CONV (*N* = 8) and; LOW‐HCHF (*N* = 6). Details on the chemical compositions of all feed ingredients used and daily intake of digestible energy and digestible crude protein are tabulated in Table S1 and S2, respectively. All animals had ad libitum access to water and a vitamin‐mineral supplement at all times. At 2½ years of age (adulthood), all sheep had developed adiposity, and were euthanized by exsanguination following intravenous administration of Propofol (B. Braun, Melsungen, Germany; 5–6 mg/kg body weight [BW]). All tissues were isolated immediately after euthanization, total tissue weight determined (except for EPI) and samples were taken for histology and genes expression analysis. SUB and EPI samples were dissected above the *m. longissimus dorsii* and from the anterior surface of the heart, respectively, whereas PER and MES were sampled randomly after separation from their respective organs (Khanal et al., 2020).

### 2.2 Tissue preparation and histology

Adipose tissue specimens were fixed in 4% paraformaldehyde (PFA) solution for 24 hr and in by 2% PFA for another week. Before embedding, tissues were trimmed and immersed in a 70% ethanol solution for 24 hr. Sections were cut at 5 µm and subsequently stained. Epicardial adipose tissue sections were hematoxylin and eosin (HE) stained, which gave the best color differentiation of individual adipocytes in this tissue, whereas the other adipose tissues were stained with iron‐hematoxylin to provide a more marked coloring of cell membranes. The slides were scanned at 5x magnification using an automated slide scanner for bright field and fluorescence (AXIO Scan.Z1; Zeiss; Germany).

### 2.3 Histomorphometric and adipocyte size distribution analyses

The cell membrane, shape, and size of adipocytes were identified and measured using a specially designed morphometric application, Iron Hematoxylin Adipose Tissue (APP ID 10113; Visiopharm^®^; Figure S1). This application consists of three protocols, where the first calculates the relative percentages of different tissue structures in the slide, that is, adipocytes, cell membranes, and undefined area. The second protocol classifies the shape of individual adipocytes according to a “Form Factor” (Figure S1a) ranging from 0 (being a straight line) to 1 (being a perfect circle). The third protocol calculates the cross‐sectional area (CSA) of individual adipocytes (Figure S1b) and automatically categorizes adipocytes into cell size classes ranging from 0–40, 40–200, 200–400, 400–800, 800–1,600, 1,600–3,200, 3,200–6,400, 6,400–12,800, 12,800–26,500, 26,500–36,000, and >36,000 µm^2^. The cell size class >36,000 µm^2^ was not included in calculations, since it contained a high proportion of cells with inadequately stained membranes, that is, the program could not identify the individual cells. The average CSA for cells determined on the slides will expectedly be lower than the average diameter of cells measured at their centers because cells in slides were cut at varying distances from their center. However, changes in distribution patterns are expected to reflect differences in the size of cell populations. A cell number index (CNI; arbitrary units) was calculated as previously described (Khanal et al., 2020):

CNI=(adipose mass(kg)×percentage adipocyte coverage in tissue slides)/volume of an average spherical adipocyte

The volume of spherical adipocyte was calculated using the formula:

$$V\;=\;4/3\pi r^{3}$$

where the radius, *r*, was derived from a circle with the same area as the average CSA of adipocytes. Cell size distribution patterns, average CSA, and CNI allowed us to evaluate, whether differences in fat deposition resulted from changes in adipocyte numbers or size.

### 2.4 Gene expression analysis

Samples of adipose tissues were preserved in RNA*later* ^®^ Solution (Ambion, The RNA Company) for 24 hr, thereafter the solution was discarded and tissue samples then stored at −80°C pending mRNA expression analysis, conducted as previously described (Khanal et al., 2020). In short, total RNA was isolated by homogenizing approximately 150 mg tissue (TissueLyser II, QIAGEN) in 1,000 µl TRIzol® reagent (Invitrogen, Life Technologies). Phase separation was performed using 120 µl chloroform. Then, an upper aqueous phase was mixed with 500 µl isopropanol to facilitate the precipitation of RNA. The SV Total Isolation System (Promega Corporation) was used to further extract the RNA following the manufacturer's protocol except that instead of 100 µl, 50 µl nuclease‐free water was used to elude RNA. The concentration and integrity of isolated RNA was measured using NanoDrop ND‐1000 Spectrophotometer (NanoDrop Products) and Agilent 2100 Bioanalyzer (Agilent Technologies), respectively. The cDNA synthesis was performed by reverse transcription (RT) by applying Promega Reverse Transcription System (Promega). A total of 25 µl reaction mixture was prepared by adding 16.3 RNA sample and 8.7 µl master mix, respectively. The master mix (Promega) consisted of 5 µl M‐MLV 5 × Reaction Buffer, 1.3 µl dNTP Mix, 0.2 µl Random Primers, 0.4 µl Oligo (Dt) 15 Primer, 0.8 µl RNasin Ribonuclease Inhibitor, and 1 µl M‐MLV reverse transcriptase. The cDNA samples were stored at −20°C pending analysis. The qPCR was performed using a Lightcycler 480 SYBR Green I Master (Roche Diagnostic GmbH) following the manufacturer's protocols and, β‐actin (*ACTB*) was used as a reference gene. The PCR conditions used were denaturation (95°C for 10 s), annealing (60°C for 10 s), and elongation (72°C for 20 s), which were repeated 45 times in each qPCR reaction.

The genes examined involved target genes for adipogenesis (*ADIPOQ*, *CD34*, *CD44*, *CEBPB*, *PGC1A, PPARA*, *PPARG*, *PREF1, TGFB1*, and *WNT5A*), angiogenesis (*VEGF*, and *VEGFA*), lipid metabolism (*ATGL*, *CGI58*, *FABP4*, *FAS*, *HSL*, *LPL*, and *PLIN1*), glucose metabolism (*FBPASE, GAPDH*, *GLUT1*, and *GLUT4*), hormone signaling (*ADRA1*, *ADRβ1*, *GcR*, *IGF1R*, *IRS1*, and *LEPTIN*), energy homeostasis and obesity (*FTO*), mitochondrial‐derived reactive oxygen species synthesis (*UCP2*), as well as markers for inflammation (*CD68*, *IL6, MCP1*, *TLR4*, and *TNFA*). The primer sequences and efficiencies are listed in Table S3.

### 2.5 Statistical analysis

Data were analyzed separately for each adipose tissue by linear mixed effects (nlme) (version 3.1–137), and emmeans (v1.3.3 and v1.4.5) (2019) procedures of the R studio (R Core Team, 2017) software package using the following overall model:

$$Y=PreN+PostN+Sex+PreN*PostN+PreN*Sex+PostN*Sex+BWewe+BWbirth+BW6+BW2\frac{1}{2}$$

where *Υ* is the observed dependent variables, *PreN* is the fixed effect of prenatal nutrition (NORM, LOW, HIGH*, PostN* is the fixed effect of postnatal nutrition (CONV, HCHF)*, Sex* is the fixed effect of sex (♂=males, ♀=females) on adipose morphological traits, adipocyte size distribution, and mRNA expression. Body weights of the twin‐pregnant dams at the onset of the experiment 6 weeks prepartum (*BWewe*) and of the experimental sheep at birth (*BWbirth*), at six months of age, that is, when the differential postnatal feeding ended (*BW6*), and by the end of the experiment in adulthood at 2½ years of age (*BW2*½) were included as covariates in the models. The normality of the model was examined using the Shapiro‐Wilk's test and qqnorm plots of the residuals. If the model did not follow a normal distribution, the model was normalized by log‐transformation. The stepwise model reduction was further performed using package MASS (v7.3–51.5) and the model having the lowest AIC was selected for the best‐fit model. *Post hoc* analysis was performed using Tukey's multiple comparison tests, when one of the main effects or their interactions were significant. Data are presented as emmean ± *SEM*, and *p*‐values provided are based on ANOVA. Correlations within different adipocyte size classes and between adipocyte size classes with gene expression levels were examined using R package “corrplot” (Version 0.84; Wei & Simko, 2017), and Pearson correlation coefficients were derived from correlation plots.

## 3 RESULTS

This study was, as mentioned above, part of a larger experiment. To be able to put the results of the present into perspective, some previously reported findings from the same animals are briefly mentioned here. LOW sheep were born with lower birth weights compared to HIGH and NORM sheep (Khanal et al., 2020). Compared to NORM, LOW as well as HIGH sheep deposited a greater amount of fat in MES and PER than SUB, when they became obese as adolescents upon exposure to the HCHF diet (Khanal et al., 2020). At the age of 2½ years, all sheep had been exposed to the same low‐fat hay‐based diet for 2 years; LOW‐HCHF sheep (99.0 ± 2.7 kg) became heavier as adults compared to NORM‐CONV and LOW‐CONV sheep (91.13 ± 2.9 and 91.2 ± 2.4 kg, respectively) with others groups in between (Table S4; Khanal et al., 2020). Males were heavier (99.4 ± 1.2 kg) than females (92.9 ± 1.4 kg; Khanal et al., 2020) The adult LOW‐HCHF sheep had markedly increased plasma levels of cholesterol, urea, creatinine, and lactate compared to other groups (Khanal et al., 2016).

### 3.1 Overall tissue‐ and sex‐specific differences in adipose cell size distribution

**FIGURE 2:**
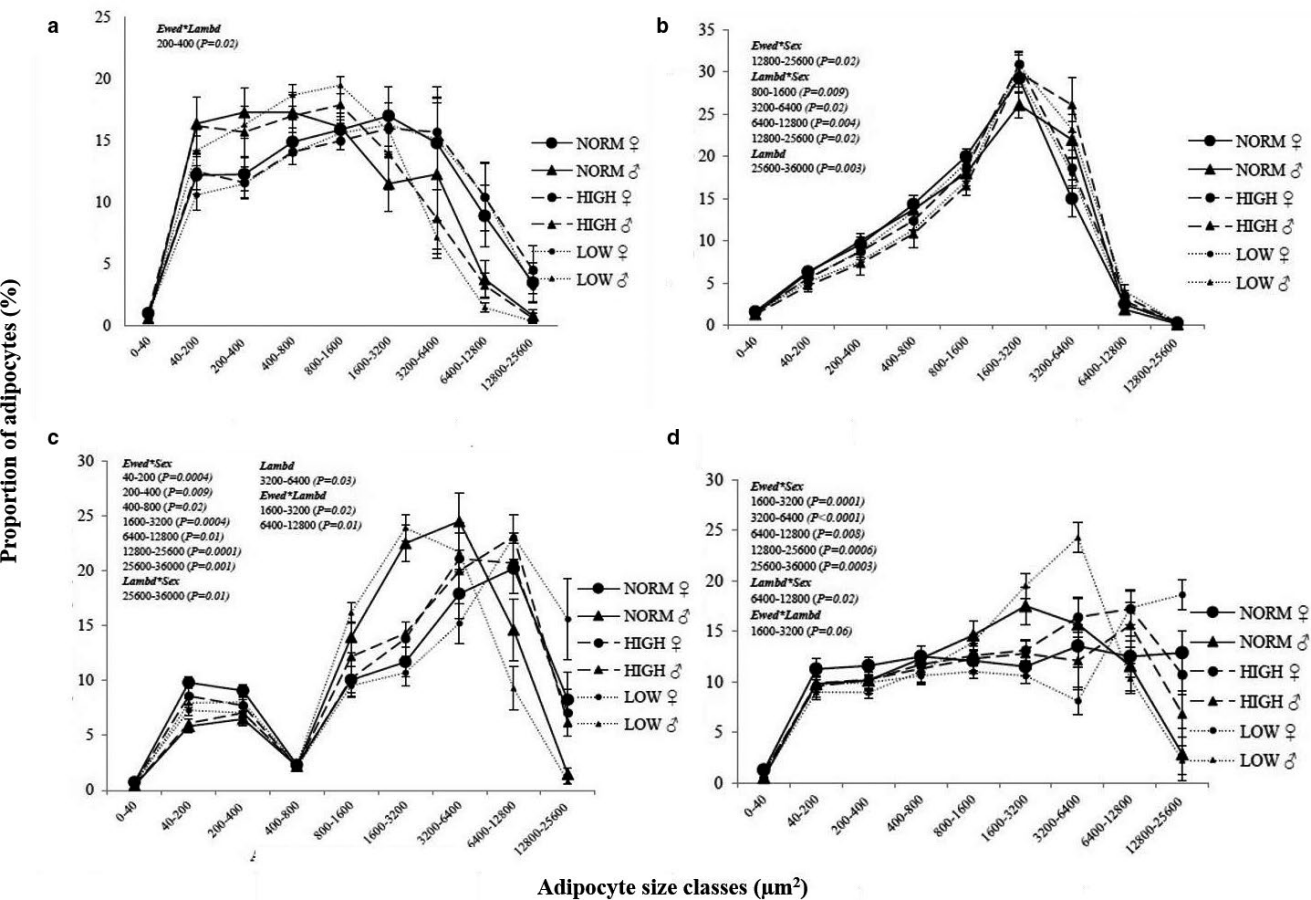
Effects of prenatal and early postnatal nutrition on the adipocyte size distribution patterns in tissue slides of (a) subcutaneous (SUB), (b) epicardial (EPI), (c) perirenal (PER) and (d) mesenteric (MES) adipose tissue from male (♂) and female (♀) 2½ years old adult sheep. Values are expressed as the proportion (%) of adipocytes found in different cell size classes. Tissue slides were stained with Iron‐Hematoxylin except for EPI, which was stained with Hematoxylin and eosin for optimal staining of cell membranes. Cross‐sectional area of individual cells was determined in whole tissue scans using the Iron Haematoxylin Adipose Tissue software (APP ID 10,113; Visiopharm^®^, Hoersholm, Denmark). NORM, HIGH, LOW, CONV and HCHF: See legends to Figure 1. Number of animals in the different groups: NORM (*N* = 9, 3♂:6♀); HIGH (*N* = 12, 5♂:7♀) and; LOW (*N* = 15, 8♂:7♀). Number of animals in the early postnatal nutrition groups: CONV (*N* = 20, 8♂:12♀) and HCHF (*N* = 17, 8♂: 8♀). Ewed, Lambd, sex, Ewed*sex, Lambd*sex, Ewed*Lambd indicate significant effects found of the late gestation nutrition, early postnatal nutrition, sex of the sheep or their interactions, respectively

In the present part of the study, distribution of adipocytes on size classes was found to follow a unimodal pattern in SUB and EPI (Figure 2a,b), whereas a clear bimodal pattern was observed in PER with the second peak falling in different cell‐size classes depending on treatment group (Figure 2c). In MES, there was a plateau across the lower (40–800 µm^2^) cell‐size classes, which continued in NORM♀ (unimodal pattern) or was followed by a more or less clear second peak in the other groups (bimodal pattern; Figure 2d).

As shown in Tables 1, 2 and Figure 2, females had the highest fat mass (not determined in EPI), adipocyte CSA (except for HIGH♀ in PER), CNI (PER only), and proportions of very small (<40 µm^2^; PER and MES only) and large adipocytes (SUB: >3,200 µm^2^; MES and PER: >6,400 µm^2^), but lowest proportions of smaller‐ to medium‐sized adipocytes (SUB: 40–1,600 µm^2^, PER: 800–6,400 µm^2^; MES: 800–3,200 µm^2^). However, in EPI, only few sex effects were seen, and only as interactions with the postnatal nutrition (see below).

**TABLE 1:** Effects of prenatal nutrition and sex on the morphological composition of adipose tissues in sheep

	Prenatal nutrition						*p*‐value					
	**NORM**		**HIGH**		**LOW**							
Sex	♂	♀	♂	♀	♂	♀	Ewed* **Sex**	**Ewed**	Lambd* **Sex**	**Lambd**	**Sex**	Ewed* **Lambd**
SUB												
Average CSA	1691.0 ± 622.0	3,171.0 ± 439.0	1,470.0 ± 656.0	3,862.0 ± 442.0	1649.0 ± 337.0	3,113.0 ± 329.0	0.57	0.38	0.90	0.94	0.0001	0.54
CNI	4.1 ± 1.7	5.5 ± 1.2	4.4 ± 1.8	3.9 ± 1.2	4.1 ± 0.9	5.9 ± 0.9	0.70	0.78	0.98	0.99	0.28	0.42
Membrane area	34.7 ± 3.1	26.9 ± 1.6	33.8 ± 3.2	25.4 ± 1.5	36.8 ± 1.8	25.7 ± 1.3	0.78	0.24	0.94	0.23	<0.0001	0.25
Cell area	64.5 ± 2.8	73.4 ± 2.0	65.7 ± 3.0	74.5 ± 1.9	62.9 ± 1.5	74.2 ± 1.6	0.86	0.21	0.86	0.25	<0.0001	0.20
Undefined area	14.7 ± 4.3^ab^	11.0 ± 1.9^ab^	11.6 ± 2.7^ab^	11.5 ± 1.9^ab^	21.7 ± 3.6^b^	7.0 ± 1.2^a^	0.03	0.92	0.88	0.16	<0.0001	0.04
PER												
Average CSA	3,709.0 ± 532.0^ab^	5,109.0 ± 425.0^bc^	4,926.0 ± 517.0^bc^	4,580.0 ± 434.0^b^	2,842.0 ± 373.0^a^	6,355.0 ± 359.0^c^	0.002	0.77	0.51	0.05	<0.0001	0.16
CNI	3.0 ± 0.8	7.4 ± 0.6	5.00 ± 0.8	7.7 ± 0.7	5.3 ± 0.6	6.5 ± 0.6	0.08	0.34	0.70	0.34	0.004	0.73
Membrane area	26.0 ± 1.6^ab^	23.8 ± 1.3^ab^	23.3 ± 1.5^ab^	23.1 ± 1.2^a^	29.1 ± 1.1^b^	20.8 ± 1.1^a^	0.02	0.31	0.47	0.10	<0.0001	0.11
Cell area	74.0 ± 1.6^ab^	76.2 ± 1.3^ab^	76.7 ± 1.5^ab^	76.9 ± 1.2^b^	70.9 ± 1.1^a^	79.2 ± 1.1^b^	0.02	0.31	0.47	0.10	<0.0001	0.12
Undefined area	4.3 ± 1.2	7.2 ± 1.7	5.0 ± 1.7	5.1 ± 1.2	6.2 ± 1.2	5.5 ± 1.1	0.32	0.81	0.42	0.26	0.17	0.88
MES												
Average CSA	3,192.0 ± 697.0	5,218.0 ± 569.0	4,025.0 ± 623.0	4,616.0 ± 569.0	3,441.0 ± 493.0	6,103.0 ± 493.0	0.18	0.69	0.59	0.57	0.0003	0.54
CNI	10.2 ± 3.3	15.3 ± 3.0	12.5 ± 4.3	15.0 ± 2.9	8.8 ± 2.3	11.9 ± 2.2	0.84	0.24	0.99	0.21	0.18	0.73
Membrane area	27.1 ± 1.8^bc^	21.0 ± 1.2^ab^	22.8 ± 1.4^abc^	21.6 ± 1.1^ab^	26.9 ± 1.1^c^	19.2 ± 0.9^a^	0.02	0.54	0.26	0.85	<0.0001	0.07
Cell area	72.4 ± 1.7^ab^	78.9 ± 1.4^bc^	77.1 ± 1.5^abc^	78.3 ± 1.3^bc^	72.7 ± 1.0^a^	81.1 ± 1.1^c^	0.01	0.40	0.22	0.69	<0.0001	0.05
Undefined area	4.60 ± 1.39	4.14 ± 0.92	4.33 ± 1.13	5.75 ± 1.20	9.06 ± 1.70	4.51 ± 0.87	0.08	0.32	0.32	0.34	0.06	0.49
EPI												
Average CSA	2,197 ± 119	1947 ± 108	2,422 ± 112	2,172 ± 104	2,437 ± 94	2,187 ± 98	0.62	0.11	0.06	0.03	0.09	0.55
Membrane area	33.10 ± 1.00^b^	28.90 ± 0.70^a^	27.80 ± 0.80^a^	27.50 ± 0.60^a^	26.40 ± 0.60^a^	28.70 ± 0.60^a^	0.01	0.001	0.13	0.90	0.49	0.06
Cell area	66.90 ± 1.00^a^	71.10 ± 0.70^b^	72.20 ± 0.80^b^	72.50 ± 0.60^b^	73.60 ± 0.60^b^	71.30 ± 0.60^b^	0.01	0.002	0.13	0.90	0.49	0.06
Undefined area	5.55 ± 0.95	5.16 ± 0.82	3.35 ± 0.73	5.05 ± 0.75	6.06 ± 0.70	4.95 ± 0.62	0.18	0.22	0.11	0.01	0.15	0.50

Note

NORM, HIGH, LOW, CONV, HCHF, ♂, ♀, Ewed, Ewed*Sex, Lambd, Lambd*Sex and, Ewed*Lambd: see legends to Figure 1. The values are expressed as emmean ± *SEM* derived from proportions (%) with 95% confidence interval. The effects were significant *p* < .05. ^abc^ Significant differences between groups are denoted by different superscript letters within row. Number of animals per groups are; SUB: NORM (*N* = 9, 3♂:6♀); HIGH (*N* = 12, 5♂:7♀); LOW (*N* = 15, 8♂:7♀). PER: NORM (*N* = 10, 4♂:6♀); HIGH (*N* = 12, 5♂:7♀); LOW (*N* = 15, 8♂:7♀). MES: NORM (*N* = 10, 4♂: 6♀); HIGH (*N* = 12, 5♂:7♀); LOW (*N* = 15, 8♂:7♀). EPI: NORM (*N* = 10, 4♂:6♀); HIGH (*N* = 12, 5♂:7♀); LOW (*N* = 15, 8♂:7♀). Values for effects of the early postnatal nutrition are presented in Tables S2 and S3.

**TABLE 2:** Effect of pre and early postnatal nutrition on the weight of adipose tissues in sheep

	Prenatal nutrition											
	**NORM**		**HIGH**		**LOW**		**P‐Value**					
Sex:	**♂**	**♀**	**♂**	**♀**	**♂**	**♀**	Ewed* **Sex**	**Ewed**	Lambd* **Sex**	**Lambd**	**Sex**	Ewed* **Lambd**
Tissue weight (g)												
SUB	241 ± 59	1,242 ± 283	292 ± 89	875 ± 183	229 ± 37	934 ± 146	0.40	0.52	0.73	0.83	<0.0001	0.36
PER	707 ± 291^a^	2,682 ± 246^bc^	1708 ± 262^ab^	2,330 ± 237^bc^	926 ± 205^a^	3,014 ± 209^c^	0.008	0.81	0.97	0.02	<0.0001	0.10
MES	2,199 ± 533^ab^	4,719 ± 447^c^	2,997 ± 590^abc^	4,125 ± 450^bc^	1746 ± 390^a^	4,661 ± 370^c^	0.22	0.41	0.69	0.09	<0.0001	0.69

Note

NORM, HIGH, LOW, CONV, HCHF, ♂, ♀, Ewed*Sex, Ewed, Lambd*Sex, Lambd, Sex, and Ewed*Lambd: see legends to Figure 1. The values are expressed as emmean ± *SEM* derived from proportions (%) with 95% confidence interval. ^abc^ Significant differences between groups are denoted by different superscript letters within row. Number of animals per groups are as follows: NORM (*N* = 10, 4♂:6♀); HIGH (*N* = 11, 5♂:6♀) and; LOW (*N* = 16, 8♂:8♀). For the lambd effect, HCHF (1793 ± 86g) animals had less PER mass than CONV (198 ± 85 g) animals.

In all tissues, positive correlations existed between adipocytes numbers within the smaller cell‐size classes (SUB and MES: 200–1,600 µm^2^, *p* < .001 and *p* < .001–.05; PER: 200–3,200, *p* < .01–.001 µm^2^; EPI: 40–1,600 µm^2^, *p* < .001–.05) and within the large cell‐size classes (SUB: 3,200–36,000 µm^2^, *p* < .001–.01; PER and MES: >12,800 µm^2^, *p* < .001–.01; EPI: >6,400 µm^2^, *p* < .001–.05). Correlations between the small and large cell size classes were generally negative (Figure S2a–e), except for the very smallest (<40 µm^2^) adipocytes in PER and MES, which (unlike other small cells) were positively correlated with numbers of the largest adipocytes (PER: 12,800–36,000 µm^2^, *p* < .001; MES: >36,000 µm^2^, *p* < .05; Figure S2b and S2c).

### 3.2 Long‐term impacts of early nutrition history on adipose tissue histology

Implications of malnutrition in early life on adipose morphology and gene expression patterns were, in general, more predominant in males than females, but in a tissue‐dependent way.

### 3.3 Subcutaneous adipose tissue

There were generally no prolonged effects of early life nutrition history on SUB mass, CNI, average adipocyte CSA (Table 1) or adipocyte size distribution (Figure 2a). The only exceptions were for very small adipocytes (<40 µm^2^), where NORM♀ and HIGH♂ had a higher percentage compared to NORM♂ and with other groups in between (*p* = .01). The proportion of 40–200 µm^2^ adipocytes was increased by HCHF compared to CONV in HIGH, but decreased by HCHF in NORM and LOW sheep (Table S4; *p* = .02).

### 3.4 Perirenal adipose tissue

This was the adipose tissue most affected by the prenatal nutrition history, but in a sex‐specific way. LOW♀ had the highest and LOW♂ (followed by NORM♂) had the lowest PER mass, average CSA of adipocytes and adipose cell coverage (*p* = .01, 0.002, and 0.01, respectively) with other groups in between.

In the first cell size peak (40–400 µm^2^), NORM♀ had the highest and NORM♂ the lowest ratio of adipocytes, (*p* < .01). In the second cell size peak, the NORM♂ and LOW♂ peaked earlier and had a higher ratio of cells in the 1,600–6,400 µm^2^ classes and lowest proportions of cells in the largest adipocyte classes, whereas HIGH♂ together with all females peaked in the 6,400–12,800 µm^2^ cell size classes (*p* < .01). LOW♀ had a markedly higher proportion of large adipocytes (>12,800 µm^2^) (*p* < .001) compared to other groups. Interestingly, HIGH♂, unlike NORM♂ and LOW♂, had a phenotype similar to female sheep for most of the studied parameters (adipocyte CSA, tissue composition, and cell size distribution).

Surprisingly, sheep fed the HCHF diet in early postnatal life had a lower adult PER mass than CONV sheep (*p* = .02; Table 2). This was associated with a slightly smaller adipocyte average CSA and a lower proportion of the largest (25,600–36,000 µm^2^), but increased proportion of medium‐sized adipocytes (3,200–6,400 µm^2^) in HCHF compared to CONV (*p* = .05, 0.01, and 0.03, respectively, Table S5). For medium to large adipocytes (6,400–25,600 µm^2^), the proportion was increased by HCHF in NORM and LOW sheep, but decreased by HCHF in HIGH sheep (*p* = .01 and .005; Table S4). The CNI was not affected by the early life nutrition history (Table 1).

### 3.5 Mesenteric adipose tissue

Impacts of the prenatal nutrition history were sex‐specific. Hence, LOW♂ and NORM♂ had clear peaks in the lower cell size classes (1,600–3,200 and 3,200–6,400 µm^2^) and the lowest proportion of the largest adipocytes compared to other animals; the adipocyte distribution pattern for HCHF♂ was very similar to that of females rather than other males, and in the largest cell size classes (>12,800 µm^2^), LOW♀ had by far the highest proportions of adipocytes and with LOW♂ at the other extreme (lowest proportion; Figure 2d; *p* = .00001–.008).

There were no systematic long‐term impacts of the early postnatal nutrition exposure in this tissue.

### 3.6 Epicardial adipose tissue

The EPI was, in contrast to the other tissues, strongly affected by the early postnatal nutrition history with hardly any effects of prenatal nutrition (Table 1). HCHF sheep had larger average adipocyte CSA (Table S5) and a shift in cell size distribution (Figure 2b) toward higher proportions of both small (40–200 µm^2^; *p* = .01) and large (>6,400 µm^2^; *p* = .003 to <.0011) at the expense of medium‐sized adipocytes (1600–3,200 µm^2^; *p* < .0001). The HCHF diet increased and decreased proportions of medium to large (3,200–6,400 µm^2^) and medium (800–1,600 µm^2^) sized adipocytes, respectively, in females, whereas males had the opposite responses to HCHF (*p* = .02 and .01, respectively).

### 3.7 Correlations between cell‐number‐index and numbers of very small (<40 µm^2^) and very large adipocytes (25000–36000 µm^2^) across tissues

Within SUB and MES, CNI was negatively correlated with adipocyte numbers in the largest cell size class (25,600–36,000 µm^2^; *r *= −.39 and −.31, respectively; *p* < .05; Figure S2e). Across tissues, CNI in PER was positively correlated with numbers of very small adipocytes in SUB (*r* = .37, *p* < .05) and to numbers of very large adipocytes in SUB (*r* = .42, *p* < .05) and MES (*r* = .48, *p* < .01). Numbers of very large adipocytes in MES were positively correlated with numbers of very small adipocytes in PER (*r* = .28, *p* < .05) and to very large adipocytes in SUB (*r* = .33, *p* < .05) and PER (*r* = .50, *p* < .01).

### 3.8 Systematic sex differences in mRNA expression levels

Across the four tissues studied, males had consistently and markedly higher expression levels than females for almost all genes in all adipose tissues (Figure S3), although males only had around half or less fat mass in these tissues (not determined in EPI) compared to females (Table 2). The only genes, where females had the highest expression level, were *LPL* in SUB and EPI, *LEPTIN* and *CD68* in PER and *CGI58* in EPI.

### 3.9 Long‐term impacts of the early nutrition history on mRNA expression levels

The *ADRB1, FTO,* and *LEPTIN* were the only genes, for which expression levels were completely unaffected by the early life nutrition history.

### 3.10 Subcutaneous adipose tissue

**FIGURE 3:**
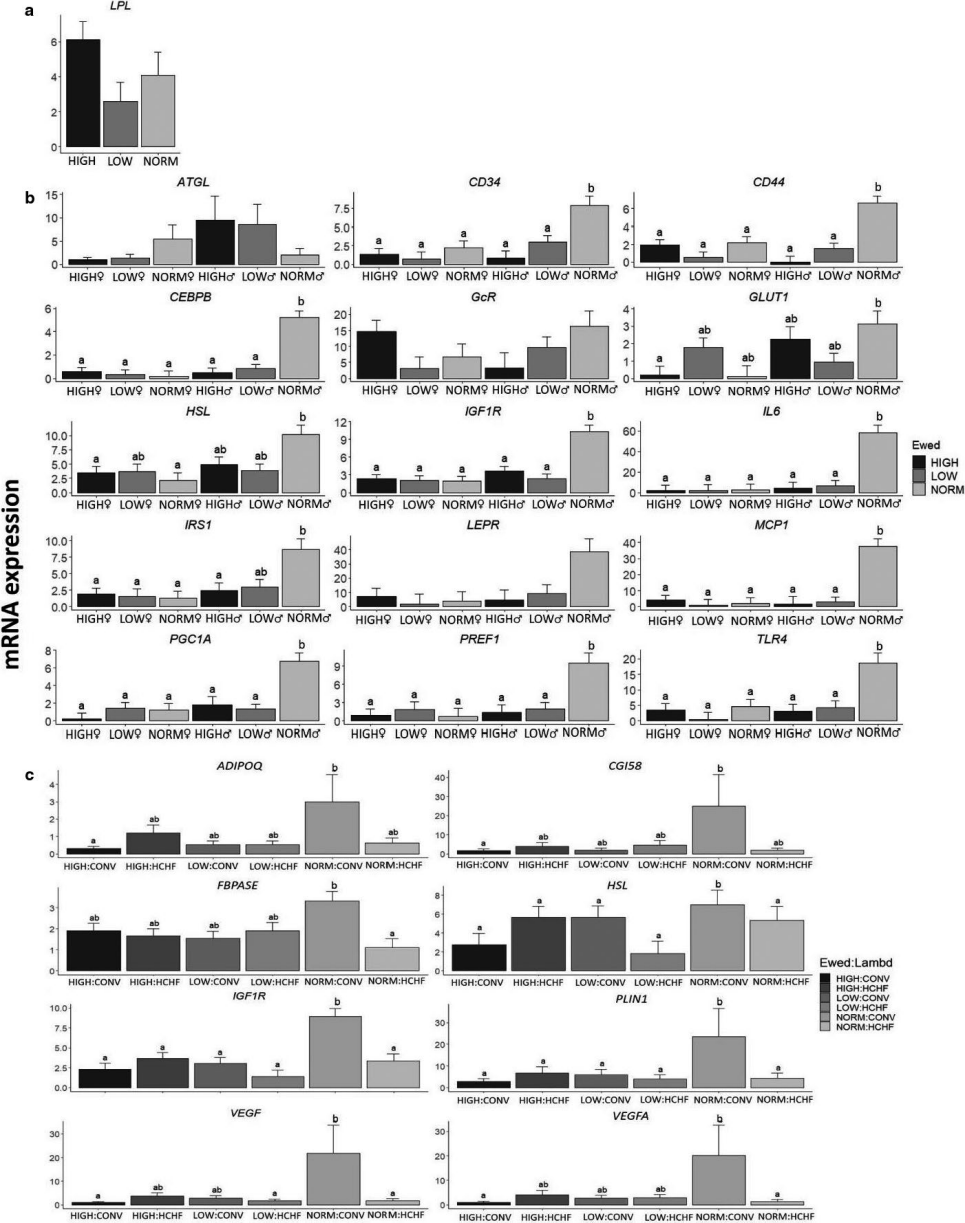
Effects of prenatal nutrition (a), sex‐dependent effects of the prenatal nutrition (b) and the interactive effects of pre‐ and early postnatal nutrition (c) on mRNA expression patterns in subcutaneous adipose tissue (SUB), expressed relative to the reference gene, beta‐actin (*ACTB*). NORM, HIGH, LOW, ♂, ♀, Ewed and sex: See legends to Figures 1 and 2. Values are expressed as emmean ± *SEM*. ^ab^ Significant differences between groups are denoted by different superscript letters. Number of animals in the prenatal nutrition groups: a) NORM (*N* = 8, 3♂:5♀); HIGH (*N* = 10, 5♂:5♀) and; LOW (*N* = 11, 6♂:5♀). Number of animals in the pre‐ and postnatal interaction groups: NORM:CONV (*N* = 6); NORM:HCHF (*N* = 4); HIGH:CONV (*N* = 6); HIGH:HCHF (*N* = 6); LOW:CONV (*N* = 8) and; LOW:HCHF (*N* = 6)

*LPL* was the only gene affected (independently of other factors) by the prenatal nutrition, and LOW had the lowest expression levels followed by NORM and HIGH (Figure 3a; *p* = .03). The mRNA expression of 15 genes involved in adipose development and metabolism was affected by the prenatal nutrition in a sex‐specific way (Figure 3b). For all except one gene (*ATGL*), NORM♂ had higher mRNA expression levels (Figure 3b; *p* < .0001–.05), whereas for *ATGL,* the expression level was higher in HIGH♂ and LOW♂ than other groups. In females, if anything, expression levels for several genes were consistently different (higher or lower) in LOW♀ compared to NORM♀ and HIGH♀.

Regarding early postnatal nutrition, HCHF sheep had decreased expression levels for *GLUT1*, *PGC1A,* and *TNFA*, and increased *PPARG* expression (Figure S4a; *p* = .004–.03). For five other genes (*CD44*, *CEBPB*, *FBPASE*, *IL6,* and *MCP1*, Figure S4a), higher expression levels were observed in CONV♂ compared to other groups. For 15 genes (*ADIPOQ*, *CD44*, *CEBPB*, *CGI58*, *FBPASE*, *GADPH*, *HSL*, *IGF1R*, *IL6*, *IRS1*, *MCP1*, *PLIN1*, *PGC1A*, *VEGF*, and *VEGFA*) expression levels depended on the pre and postnatal interaction, and highest expression levels were observed in NORM‐CONV compared to all other groups (*p* = .003–0.05; Figure 3c). The only deviation from this pattern was that HIGH‐HCHF had the highest *LPL* expression (*p* = .02).

### 3.11 Perirenal adipose tissue

**FIGURE 4:**
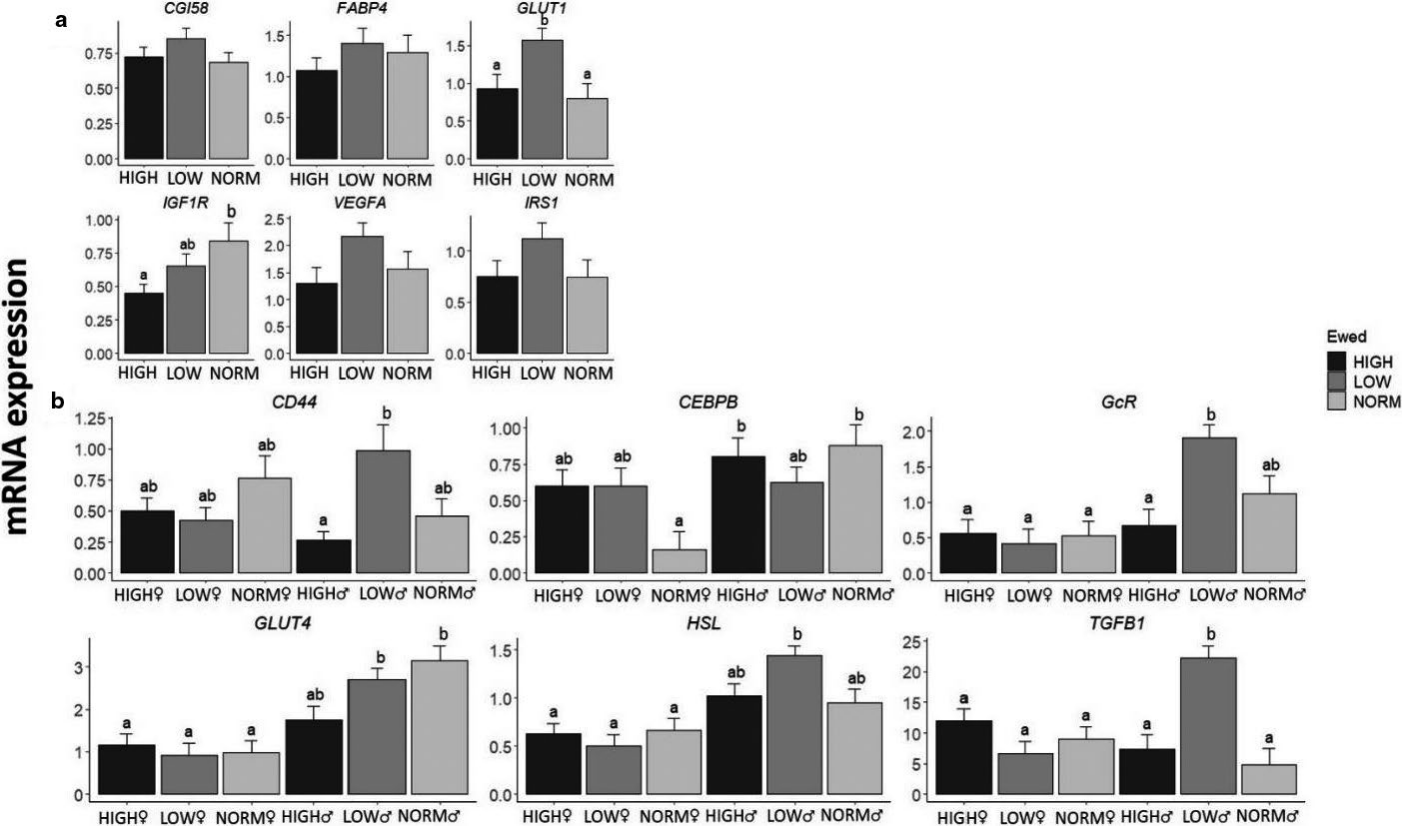
Effects of prenatal nutrition (a), sex‐dependent effects of the prenatal nutrition (b) and the interactive effects of pre‐ and early postnatal nutrition, expressed relative to the reference gene, beta‐actin (*ACTB*). NORM, HIGH, LOW, CONV, HCHF, Ewed, Lambd, Sex, ♂ and ♀: see legends to Figures 1 and 2. Values are expressed as emmean ± *SEM*. ^ab^ Significant differences between groups are denoted by different superscript letters. Number of animals in the prenatal nutrition groups: NORM (*N* = 10, 4♂:6♀); HIGH (*N* = 12, 5♂:7♀) and; LOW (*N* = 14, 5♂:6♀)

In PER, 17 genes were affected by the prenatal nutrition and six of them independently of other factors: LOW had the highest expression levels compared to other groups for *CGI58*, *FABP4*, *GLUT1*, *IRS1,* and *VEGFA,* or compared to HIGH for *IGF1R* (Figure 4a; *p* = .03–.03). For six genes, the prenatal impacts were sex‐specific (Figure 4b), where the general pattern was that LOW♂ attained the highest expression levels compared to other groups (*CD44*, *GcR*, *HSL,* and *TGFB1*) except that NORM♂ achieved the highest levels for *CEBPB* and *GLUT4* (Figure 4b; *p* = .0001–.02). For five genes (*HSL*, *IL6*, *MCP1*, *UCP2,* and *VEGF*), significant pre and early postnatal nutrition interactions were found, but no consistent patterns of changes could be deciphered across groups (results not shown).

Only three genes were affected by the early postnatal nutrition independently of other factors, and HCHF sheep had the highest expression levels for *CEBPB* and *PLIN1*, but lowest for *CD68* (Figure S4b; *p* = .01–.02). Similar to SUB, expression levels were highest in CONV♂ compared to other groups for three genes, *FABP4*, *GLUT4,* and *VEGF* (Figure S4b; *p* = .0007–.3). Females, if anything, tended to have the opposite response to the postnatal diet compared to males.

### 3.12 Mesenteric adipose tissue

The MES was the depot least affected by early life nutrition history in terms of gene expression patterns. Expression levels of only four genes were affected by the prenatal nutrition in either a sex‐specific way (*CD34,* and *CEBPB*; *p* = .01) or depending on the subsequent early postnatal nutrition exposure (*CD34*, *FABP4,* and *PPARG*; *p* = .02–.03), but it was difficult to discern any systematic pattern (results not shown). Furthermore, postnatal nutrition had an impact on expression levels of only one gene, namely *ADRA1*, where expression levels were highest in HCHF sheep (Figure S4c; *p* = .03).

### 3.13 Epicardial adipose tissue

**FIGURE 5:**
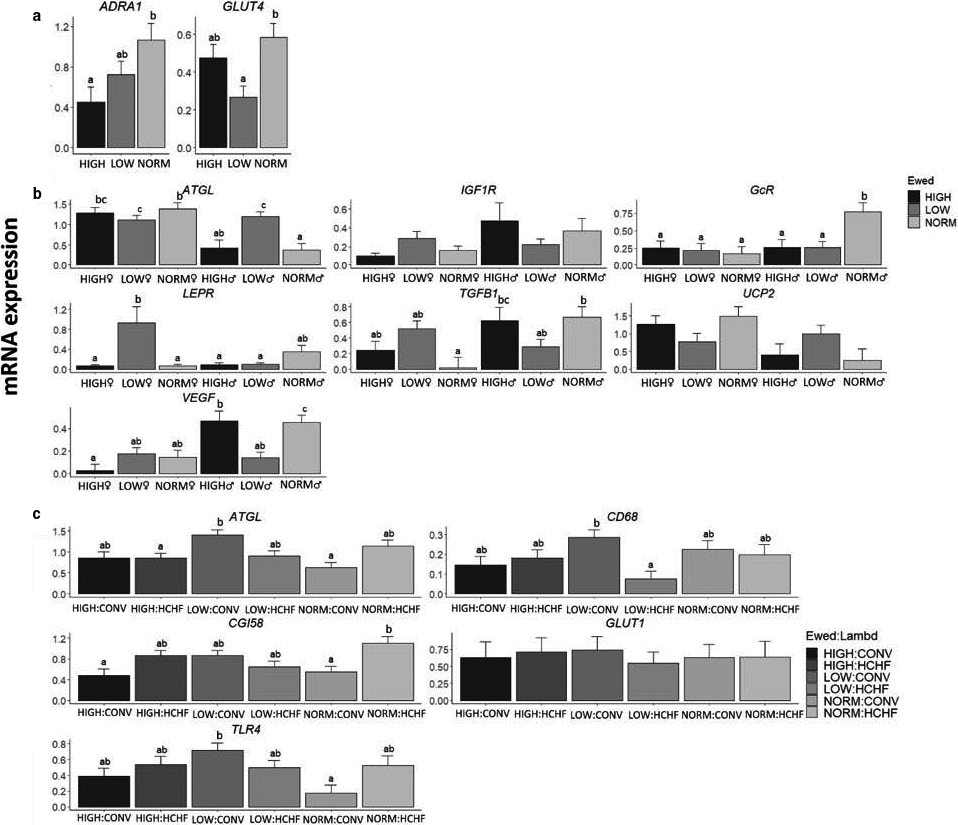
Effects of prenatal nutrition (a), sex‐dependent effects of the prenatal nutrition (b) and the interactive effects of pre‐ and early postnatal nutrition (c) on mRNA expression patterns of epicardial adipose tissue (EPI), expressed relative to the reference gene, beta‐actin (*ACTB*). NORM, HIGH, LOW, Lambd, CONV, HCHF, ♂, ♀ and Ewed, see legends to Figures 1 and 2. Values are expressed as emmean ± *SEM*. ^ab^ Significant differences between groups are denoted by different superscript letters. Number of animals in the prenatal nutrition groups: NORM (*N* = 10, 4♂:6♀); HIGH (*N* = 12, 5♂:7♀) and; LOW (*N* = 13, 8♂:7♀). Number of animals in the pre‐ and postnatal interaction group: NORM:CONV (*N* = 6); NORM:HCHF (*N* = 4); HIGH:CONV (*N* = 6); HIGH:HCHF (*N* = 6); LOW:CONV (*N* = 8) and; LOW:HCHF (*N* = 7)

The prenatal nutrition history affected the expression of 14 genes in EPI, but only independently of other factors for two genes. Thus, *ADRA1* expression levels were reduced in HIGH compared to NORM with LOW in between, and *GLUT4* expression levels were reduced in LOW compared to HIGH and NORM (Figure 5a; *p* = .04 and .02, respectively). For another seven genes (*ATGL*, *IGF1R*, *GcR*, *LEPR*, *TGFB1*, *UCP2,* and *VEGF*), the prenatal impact on expression levels was sex‐dependent due to opposite responses in LOW♂ compared to LOW♀ to the prenatal nutrition (Figure 5b).

Of all the adipose tissues, EPI was the one most affected by the early postnatal nutrition history. HCHF compared to CONV sheep had increased expression levels for 6 genes (*ADIPOQ*, *FABP4*, *FAS*, *HSL*, *PLIN1*, and *PPARG*) and reduced for 2 (*CD68*, and *PPARA*) (Figure S4d). For five genes (*ATGL*, *CD68*, *CGI58*, *GLUT1,* and *TLR4*) postnatal nutrition impacts depended on the prenatal nutrition history, and the general pattern was that LOW‐CONV had the highest expression levels for these, and the changes in expression levels induced by HCHF were opposite in LOW compared to NORM and HIGH sheep (Figure 5c; *p* = .05–.05). CONV♂ had higher expression levels compared to other groups for two genes, namely *CD34,* and *GLUT1* (Figure S4d; *p* = .04 and .01, respectively).

### 3.14 Correlation between adipocyte size and gene expression levels within each adipose depot

The significant correlations between adipocyte size classes and gene expression levels were in general weak to moderate (*r* < .6, *p* = .05–.001) in all tissues. As expected from correlations between cell size classes, it was observed that when gene expression levels were correlated with cell numbers in the smaller adipocyte classes (positive or negative), the correlation would have the opposite direction (negative or positive) to the larger adipocyte classes (Figure S5a–d). Systematic and fairly strong correlations (*r* ≥ .55, *p* = .001) between gene expression levels and adipocyte size distribution were observed almost exclusively within cell‐size classes in the second cell‐size peak in PER (see Figure 1c). Thus, adipocyte numbers in the two smallest size‐classes in the second cell‐size peak (800–1,600 µm^2^ and 1,600–3,200 µm^2^) in PER were positively correlated (*p* = .05–.001), respectively, to the expression levels of genes involved in various functions such as adipogenesis (*ADIPOQ* (*r* = .56 and .68), *CD34* (*r* = .61 and .68) and *PPARG* (*r* = .61 and .70)), lipid metabolism (*CGI58* (*r* = .54 and .63), *FABP4* (*r* = .56 and .63), and *PLIN1* (*r* = .66 and .75)), glucose metabolism (*GLUT4* (*r* = .56 and .65)), hormone signaling (*GcR* (*r* = .57 and .69), *IGFIR* (*r* = .56 and .60) and *IRS1* (*r* = .69 and .82)), energy homeostasis and obesity (*FTO* (*r* = .60 and .55)), and angiogenesis (*VEGF* (*r* = .71 and .77), and *VEGFA* (*r* = .68 and .81); Figure S5b). Opposite direction of correlations was noticed for adipocyte numbers in the medium to large (6,400–12,800 µm^2^) adipocyte size classes to expression levels for these genes (*r* = −.30 to −.7; *p* < .001–.05; Figure S5b).

Unique to PER and EPI, cell numbers in the smallest adipocyte size class (<40 µm^2^) were consistently negatively correlated in PER, but positively correlated in EPI (opposite direction of correlations), to gene expression levels of adipogenic (*PPARG* (PER: *r* = −.50, EPI: *r* = .30)), lipid metabolism (*CGI58* (PER: *r* = −.46; EPI: *r* = .38), *FBPASE* (PER: *r* = −.44, EPI: *r* = .31), *HSL* (PER: *r *= −.39, EPI: *r* = .35), *LPL* (PER: *r* = −.33; EPI: *r* = .37)), and hormone signaling (*ADRA1* (PER: *r *= −.35; EPI: *r* = .33); *p* < .01–.05) (Figure S5b,d).

## 4 DISCUSSION

This study revealed a sex‐specific upper‐limit for expandability in SUB, which was markedly lower in males than females. The major targets for prenatal malnutrition were PER and SUB, and males were more susceptible than females. LOW♂ had the lowest PER expandability, whereas HIGH♂ had an adaptive advantage due to increased hypertrophic ability, equivalent to that observed in females. MES was remarkably unaffected by the early nutrition history, and given the quite fixed expandability in SUB, our study points to PER as a major determinant of adult fat deposition patterns. EPI, in contrast to other tissues, was targeted particularly by early postnatal obesity, resulting in adipocyte hypertrophy in adulthood.

To be able to put the discussion of these findings into perspective, a brief summary is given here of previously reported findings relating to adipose tissue in a subgroup of lambs from the same experiment, which was studied as adolescents (6 months of age). The lambs exposed to LOW and HIGH nutrition in late fetal life had reduced intrinsic cellularity (CNI) in SUB and MES, reduced ability for hyperplasic growth during obesity development in SUB, PER, and MES, and a fixed upper limit for adipocyte hypertrophy in SUB. Consequently, when LOW and HIGH lambs were fed the HCHF diet in early postnatal life, expansion of PER and MES fat mass relied to a greater extent on hypertrophic rather than hyperplasic growth, and a larger proportion of fat deposition was directed toward PER and MES rather than SUB as compared to what was observed in NORM‐HCHF lambs (Khanal et al., 2020). An approximately ninefold increase in PER fat mass in HCHF compared to CONV fed lambs was associated with a 1/3 reduction in kidney size (Khanal et al., 2014).

### 4.1 General observations on adipocyte characteristics

Adipocyte size distribution in the adult sheep in this study had a unimodal appearance in SUB, a bimodal appearance in PER and MES, and a clear unimodal appearance in EPI. From studies in humans, a bimodal pattern has been reported for SUB, PER, and MES (Fang et al., 2015; McLaughlin et al., 2014), suggesting two separate cell populations. The discrepancy for SUB may be due to species differences (Karastergiou et al., 2012) or degree of adiposity. Subjects included in the two human studies were in contrast to our sheep morbidly obese, and we actually did observe an initial “shoulder” in the cell size distribution, which together with correlation structures between cell size classes indicated that two distinct cell size populations might indeed exist in this tissue also in sheep.

The CSA is expected to underestimate the actual size of adipocytes, as already mentioned, but the maximum cell sizes observed should be indicative of the CSA of the largest cells, when cut at their mid‐plane. Thus, in SUB there appeared to be a quite fixed, but sex‐specific, upper limit for adipocyte size, that is, hypertrophic expandability, irrespective of the early nutrition history, as we had also observed in lambs from the same experiment that was studied at 6 months of age (Khanal et al., 2020). This distinguished SUB from PER and MES. Subcutaneous adipocytes appear to have fixed upper limits for hypertrophy also in humans, where the average subcutaneous adipocyte sizes in obese subjects have been observed to be very similar (O'Connell et al., 2010), despite different fat distribution patterns in the body.

### 4.2 Sub and per are targets of early nutrition programming in a tissue and sex‐specific way

The quite marked reductions in intrinsic, nonobese cellularity (CNI) in SUB and MES in the adolescent (6 months old) LOW and HIGH compared to NORM lambs (Khanal et al., 2020) were not evident in the adult sheep in this study. This indicates there must have been a time window for compensatory hyperplasic expandability in these tissues after puberty, which was uncoupled from the development of obesity. This challenges a previous assumption that adipose tissue growth during most of postnatal life occurs by hypertrophy of existing adipocytes (Chusyd et al., 2016; Fang et al., 2015; Foster et al., 2011; Karastergiou et al., 2012; Lamacchia et al., 2011; Lim & Meigs, 2014; McLaughlin et al., 2014; O'Connell et al., 2010), and only in case of extreme obesity development in humans will hyperplasic cellular expansion be reactivated (Lamacchia et al., 2011) by recruitment of precursor cells (preadipocytes).

SUB adipocytes had an upper‐limit for expandability that was unaffected by the early nutrition history, but it was substantially lower in males than females, as also observed in humans (Fang et al., 2015; Karastergiou et al., 2012; O'Connell et al., 2010). This can explain the greater propensity in males for redirection of fat deposition toward visceral adipose tissues in situations of excess nutrient intake, and hence the increased risk of fat accumulation in nonadipocyte cell types (Karastergiou et al., 2012). SUB has thus been proposed to be an initiating factor in the process of redistribution of fat overflow for deposition at other sites, rather than directly implicated in the development of metabolic dysfunctions, which are caused by adipose hypertrophy associated with obesity (O'Connell et al., 2010).

Contrary to SUB, very little is known about the specific role of PER in relation to development of obesity and associated disorders, and most studies of PER in humans has relied on indirect measurements using ultrasound and other noninvasive approaches (Shuster et al., 2012). In rodents, studies on visceral adipose tissue have predominantly been conducted on epididymal fat, which does not exist in humans or sheep, and although epididymal fat possesses some characteristics similar to omental fat (Karastergiou et al., 2012), there are also dissimilarities to visceral tissues in humans (Chusyd et al., 2016). Our study points to PER as a major determinant of sex‐specific intra‐abdominal fat distribution, and a particular target of long‐term consequences of early life nutrition. NORM♂ and LOW♂ had the lowest PER fat mass among all sheep and the lowest average adipocyte CSA, in agreement with a shift in cell size distribution within the second cell size peak toward higher numbers of smaller (800–3,200 µm^2^) and lower numbers of larger (6,400–25,600 µm^2^) adipocytes compared to other groups. Quite interestingly, PER fat mass in the adult, formerly obese, HCHF sheep was quantitatively reduced compared to CONV sheep, and it was also the only tissue that had not increased several fold (and in fact numerically decreased) in weight in the adult HCHF sheep compared to the 6 months old HCHF lambs from the same experiment (Khanal et al., 2014). The underlying reason for this collapse in expandability of PER in HCHF sheep from adolescence to adulthood is unknown, but it was associated with a shift in the second cell size peak toward smaller cells similar to that described earlier for the NORM♂ and LOW♂ sheep.

Small adipocytes are often considered healthier than large hypertrophied adipocytes. When adipocytes expand above a certain size, their lipid metabolism changes resulting in the release of cytokines and potentially tissue inflammation (Stenkula & Erlanson‐Albertsson, 2018). These are risk factors in relation to the development of insulin resistance and type 2 diabetes, as reviewed (Stenkula & Erlanson‐Albertsson, 2018). However, a particular sub‐population of very small adipocytes (<40 µm^2^) in SUB and omental adipose tissues have also been shown to be associated with the degree of insulin resistance in equally obese human subjects (Fang et al., 2015; McLaughlin et al., 2007, 2014).

We observed in PER (and to a lesser extent MES), that numbers of these very small adipocytes (<40 µm^2^) were in fact positively correlated with numbers of the largest adipocytes, but negatively correlated with numbers of the other small adipocytes in cell‐size peak 1 as well as to CNI. In a normal and healthy adipose tissue, once the “threshold” for storage of fat in peak 2 adipocytes is reached, expansion of the population of smaller adipocytes is expected to occur. However, the subpopulation of very small adipocytes appears to have impaired ability to proliferate and mature into fully functional ones and/or failure in their ability to accumulate lipids (Fang et al., 2015; McLaughlin et al., 2007), and we have previously shown they may have a fetal origin in sheep (Nielsen et al., 2016).

An intriguing finding was that a HIGH level of nutrition during fetal life appeared to induce a female‐like phenotype in HIGH♂ with respect to adipocyte size distribution, and HIGH♂ had twice as much PER fat mass compared to LOW♂ and NORM♂.

Our results therefore suggest that the observed changes in cell size distribution in male (NORM and LOW) and HCHF sheep reflects a reduced expandability capacity of PER, and a HIGH level of nutrition in late fetal life offers protection against such adverse changes in males.

### 4.3 Early life nutrition impacts on morphological and gene expression changes poorly related

In the attempt to unravel the mechanisms underlying the observed alterations in adipose tissue expandability, we looked for changes in the expression of genes involved in the regulation of various (patho‐) physiological functions in adipose tissue. In SUB of NORM‐CONV and NORM♂ sheep, and in PER of LOW and particularly LOW♂, there were signs of systematic changes in gene expression compared to other groups. However, this was manifested as an upregulation of genes considered to promote adipogenesis (*CD44*, *CEBPB*), angiogenesis (*VEGF*), lipid metabolism (*CGI58*, *HSL*), glucose metabolism (*GLUT1*), and hormone signaling (*IGF1R*, *ISR1*), which was contrary to the observed changes in adipose expandability and tissue mass. In fact, NORM‐CONV and NORM♂ had the lowest SUB fat mass among all groups, and LOW♂ had the smallest PER adipocytes (CSA). Although gene expression levels could of course have adapted over time, it appears that other genetic markers than the ones included in this study must have been responsible for the observed early nutrition programing of expandability in these two tissues.

Similar to our study, a nonhuman primate (baboon) study (70% global restriction from day 30 of gestation until term) demonstrated that prenatal undernutrition induced expression of adipogenic promoting genes (such as *FABP4*) in PER of specifically males (Tchoukalova et al., 2014). This shows that the effects of maternal undernutrition are sexually dimorphic, and female fetuses appear to have a superior ability to adapt to malnutrition compared to male fetuses. In the baboon study, prenatal undernutrition was associated with hypertrophied adipocytes in omental adipose tissue of the male fetuses, in contrast to what we observed in the adult sheep. This could very well be age‐related, since PER adipocytes of adolescent LOW (and HIGH) lambs from our experiment became grossly hypertrophied, when they were fed the obesogenic HCHF diet (Khanal et al., 2020). At some point from adolescence to adulthood, there was a collapse in PER expandability in the HCHF fed.

LOW♂ in particular had increased expression levels of adipogenic genes, such as *CD44*, *TGFB1,* and HSL, and expression levels were positively correlated with numbers of adipocytes in the two smallest adipocyte classes (400–3,200 µm^2^) in the second cell size peak, whereas negative correlations were found to numbers of larger adipocytes (6,400–25,600 µm^2^) as well as to the previously mentioned population of very small adipocytes (<40 µm^2^). We therefore suggest that increased numbers of these very small and smaller peak‐2 adipocytes in PER are in fact unhealthy signs of an adipose tissue with reduced expandability and impaired metabolic function. It is tempting to speculate that the hypercholesterolemia we observed in the nutritionally mismatched adult LOW‐HCHF sheep in our study (Khanal et al., 2016) could be associated to reduce PER adipocyte expandability combined with unfavorable early programming of gene expression patterns.

The morphological signs of inflammation in SUB or PER observed in certain groups of lambs from the same experiment (Khanal et al., 2020) were not observed in the adult sheep. In agreement with this, none of the inflammatory markers was affected by the early nutrition history, except for increased expression of *MCP1* and *TLR4*, respectively, in the presumable most healthy NORM‐CONV and NORM♂ sheep. A study in 6‐week‐old obese mice, similarly demonstrated that omission of 10% fat in the diet for 12 weeks could reduce expression levels of visceral adipose tissue‐inflammatory markers (Vieira et al., 2009), and in 14‐week‐old mice, dietary intervention with a low‐fat diet or restricted (70%) high‐fat diet for 5 weeks reduced perirenal macrophage infiltration (Hoevenaars et al., 2014).

### 4.4 Secondary impacts of changes in sub and per expandability

In MES, there were sex‐specific differences in adipose morphology similar to those observed in SUB and PER. Nevertheless, it is tempting to speculate that this was not a sign of MES being a primary target of early nutrition programming, since hardly any effects on gene expression patterns could be detected. However, positive correlations were observed between numbers of large adipocytes in PER with numbers of large adipocytes in MES. Given the fixed (but sex‐specific) expandability of SUB adipocytes, the observed prenatal impacts on expandability in MES could thereby be indirect consequences of expandability changes in PER. This points to PER expandability as a major determinant for fat deposition patterns, and risk of fatty acid and cholesterol overflow into epicardial and nonadipocyte cell types in situations of nutrient excess.

Apparently, the lack of expansion of PER fat mass from adolescence into adulthood allowed for compensatory kidney growth, since no differences were observed in kidney weight between adult CONV and HCHF sheep after they had been fed the same CONV diet for 2 years. Obviously, it cannot be ruled out that the suppression of kidney growth due to massive PER expansion in HCHF lambs during the first 6 months of postnatal life may have had permanent implications for certain kidney functions. Previous studies (reviewed in Foster et al., 2011) demonstrated that the infiltration of adipocytes into the renal sinus could compress the renal vein and artery, leading to increased interstitial pressure, and excessive PER fat deposition could modify kidney function simply due to compression (Lamacchia et al., 2011). In a cross‐sectional study performed on type‐II diabetic patients (Lamacchia et al., 2011) and in the Framingham Heart Study (Foster et al., 2011), PER fat thickness was found to be a determining factor for kidney dysfunction, and individuals with “fatty kidneys” (high amount of renal sinus fat) had increased risk of hypertension and chronic kidney diseases.

In this context, it is intriguing to speculate that the hypercholesterolemia, hyperuricemia, and hypercreatinemia observed in the nutritionally mismatched adult LOW‐HCHF sheep in our study (Khanal et al., 2015) could be related to adverse programming of PER adipose tissue expandability as well as to renal dysfunctions due to the early suppression of kidney growth.

### 4.5 Epicardial adipose tissue is a target of primarily early postnatal programming

To date, scientific literature offers very limited information on the ontogenesis of EPI, and not least implications of early life malnutrition on this tissue in comparison with the previously mentioned adipose tissues. In human fetuses, EPI can be detected as early as 20–28 weeks of gestation (Jackson et al., 2016; Yavuz et al., 2016). In adults, fat surrounding the heart is associated with artery diseases independent of the amount of visceral adipose tissue (Lim & Meigs, 2014).

In our study, late gestation malnutrition had negligible impacts on adipose expandability traits and gene expression patterns in EPI in adulthood. Epidemiological studies in humans, however, have shown that a combination of maternal undernutrition in the early stage of pregnancy followed by overnutrition from day 60 up to term promoted increased the risks for adult cardiovascular diseases (Roseboom et al., 2000, 2006). Epicardial fat layer thickness was also increased in fetuses of diabetic compared to nondiabetic mothers (Jackson et al., 2016; Yavuz et al., 2016). The discrepancies between human and our sheep study are presumably due to differences in the timing of the fetal nutritional insult.

In humans, exposure to energy‐dense diets and rapid catch‐up growth in the early postnatal period is known to alter the metabolism and functionality of EPI, which may have adverse implications for cardiovascular health later in life (Twig et al., 2016; Weihrauch‐Blüher et al., 2019). Under pathological conditions, hypertrophy of epicardial adipocytes may be associated with the accumulation of lipids in the wall of the proximal coronary arteries (Iacobellis & Barbaro, 2019) and in the left atrium along the adventitia (Bornachea et al., 2018), since there is no muscle fascia separating EPI from the myocardium. In agreement with this, the obesogenic HCHF diet fed in early postnatal life in this study gave rise to adipocyte hypertrophy in EPI, which was associated with the upregulation of both adipogenic (*ADIPOQ* and *PPARG*) and lipogenic (*FAS, HSL, PLIN1*) genes. This demonstrates that EPI behaves distinctly different compared to SUB, PER, and MES, being the main target of early postnatal, but not late gestation nutritional programing. Furthermore, the upregulation of these genes in adult sheep with a history of early postnatal obesity gave rise to enlarged epicardial adipocytes, which was obviously not reversible by 2 years of dietary correction and associated correction of body fat mass later in life.

## 5 CONCLUSION

SUB had sex‐specific upper‐limits for hypertrophy, which was unrelated to the early nutrition history. Adipose cellularity, adipocyte size and hence expandability capacity of SUB, PER, and MES were markedly lower in males than females. PER was the major target of prenatal malnutrition with altered expression levels for a range of genes in LOW and specifically LOW♂, which also had reduced adipocyte hypertrophic ability. Prenatal HIGH nutrition, on the other hand, offered an adaptive advantage for males, evidenced by increased PER hypertrophic ability and expandability in HIGH♂ toward that observed in females. The genetic markers included in this study could not account for these morphological changes. With fixed upper‐limits for SUB adipocyte hypertrophy, PER expandability appears to become a major determining factor for patterns of intra‐abdominal fat deposition, rendering LOW and particularly LOW♂ predisposed for MES and nonadipose lipid accumulation with associated metabolic risks in adulthood. Contrary to the other three tissues, EPI was a major target of early postnatal obesity development, which tracked into adulthood as increased expression levels for adipogenic and lipogenic genes associated with adipocyte hypertrophy.

## CONFLICT OF INTEREST

The authors declare that they have no conflict of interest.

## AUTHOR CONTRIBUTION

MON was the principal investigator of this project. SA and MON interpreted the histomorphometric and gene expression data and were the major contributors in writing the manuscript. LKL and MM carried out the gene expression analysis. SA and MM performed the histomorphometric analysis of the adipose tissue. SA and RD performed the statistical analysis. JSA and PK contributed to the revision of the article. MON had the primary responsibility for the content of the paper. All authors read and approved the final manuscript.

## ETHICAL STATEMENT

All the experimental animal handling procedures were approved by the Danish National Committee on Animal Experimentation.

## Supporting information



Fig S1‐S5

Table S1‐S5

## Data Availability

Not applicable.
