# Supplementary material for: Depot and sex‐specific implications for adipose tissue expandability and functional traits in adulthood of late prenatal and early postnatal malnutrition in a precocial sheep model
Source: Physiol Rep. 2020 Oct 10;8(19):e14600. doi: 10.14814/phy2.14600 (PMC7547587; doi:10.14814/phy2.14600)
Supplement: Supplementary file 1 — Fig S1‐S5 [file PHY2-8-e14600-s001.docx]

**ss**

**Supplementary Figures**

**
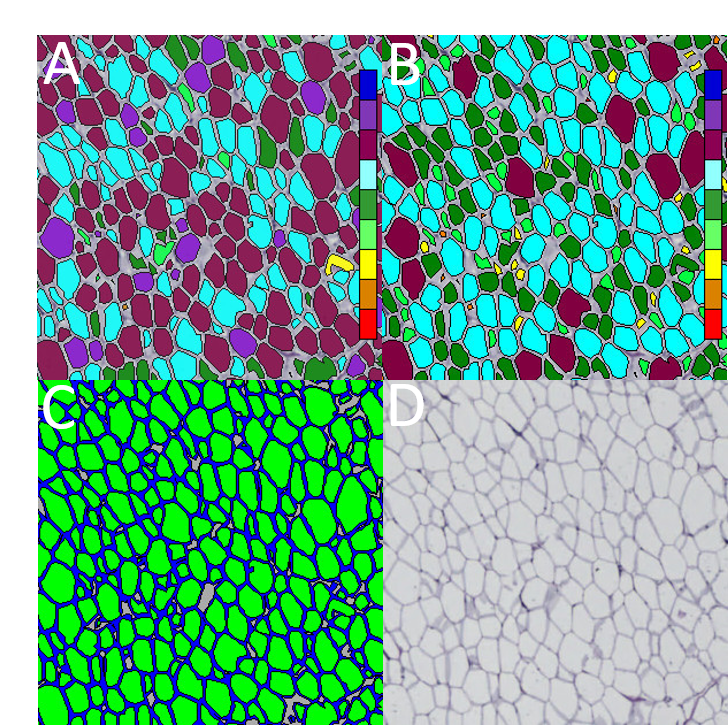
**

**Figure S1** Iron hematoxylin, Adipose Tissue App (Visiopharm^®^ software with application (APP) name; Iron Haematoxylin, Adipose Tissue (APP ID 10113), version 1.0). (A) Adipose cells classification based on Form Factor (classes range from a Form Factor value of 0 (red; straight line) to 1 (dark blue; perfect circle), as indicated by the color bar). (B) Adipose cells classification based on cross-sectional area (CSA; classes range from a CSA of 60 um^2^ (red) to 36000 um^2^ (dark blue), as indicated by the color bar). (C) Membrane and cell area detected by the APP “02 Quantify Fat Cell Membranes”. (D) Example of stained adipose tissue upon which the App is applied.

**
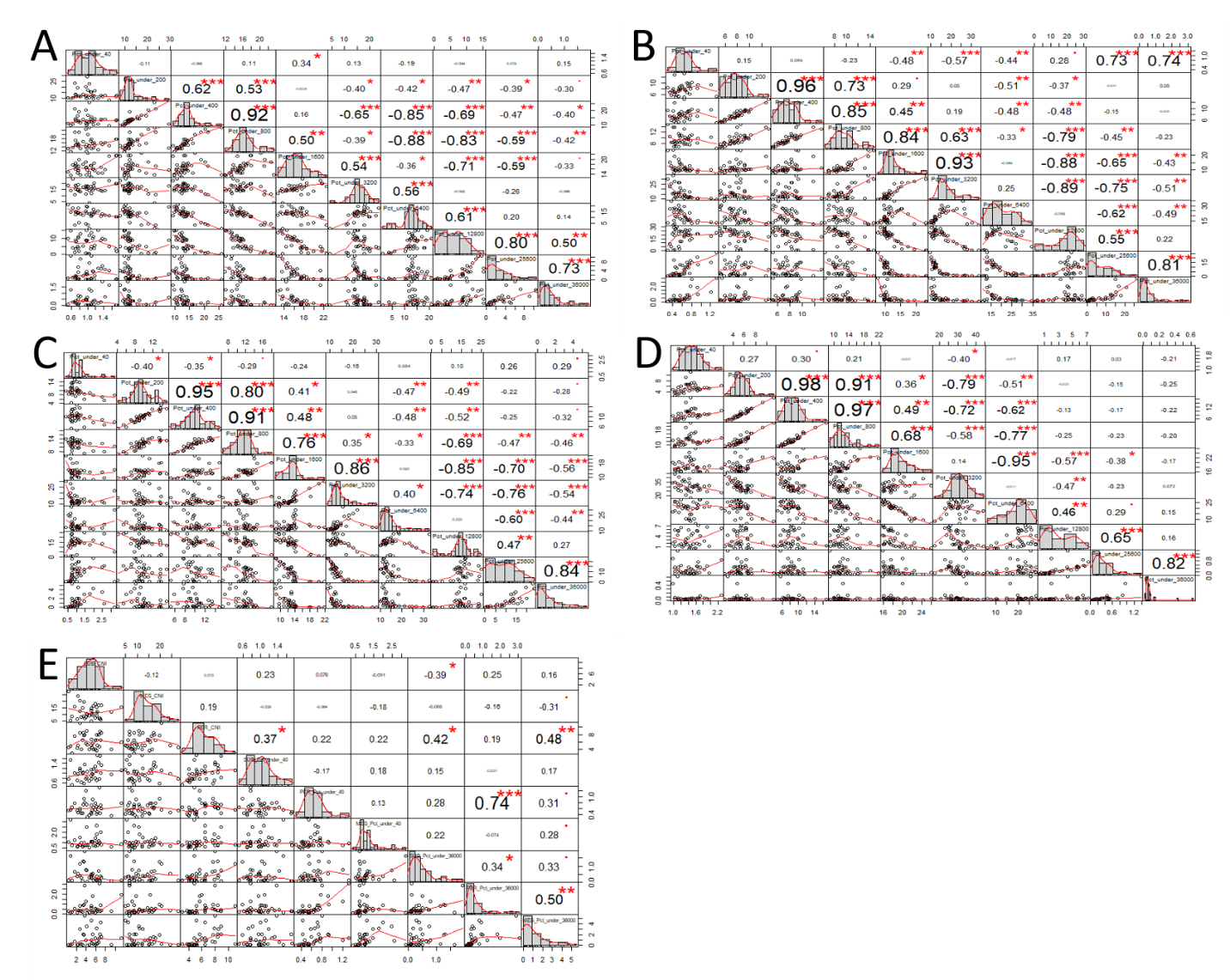
Figure S2** Overview of correlations (r) between numbers of adipocytes in different cell size classes within (A) subcutaneous, (B) perirenal, (C) mesenteric, and (D) epicardial adipose tissues from 2½ year old adult sheep with different early nutrition histories.

**
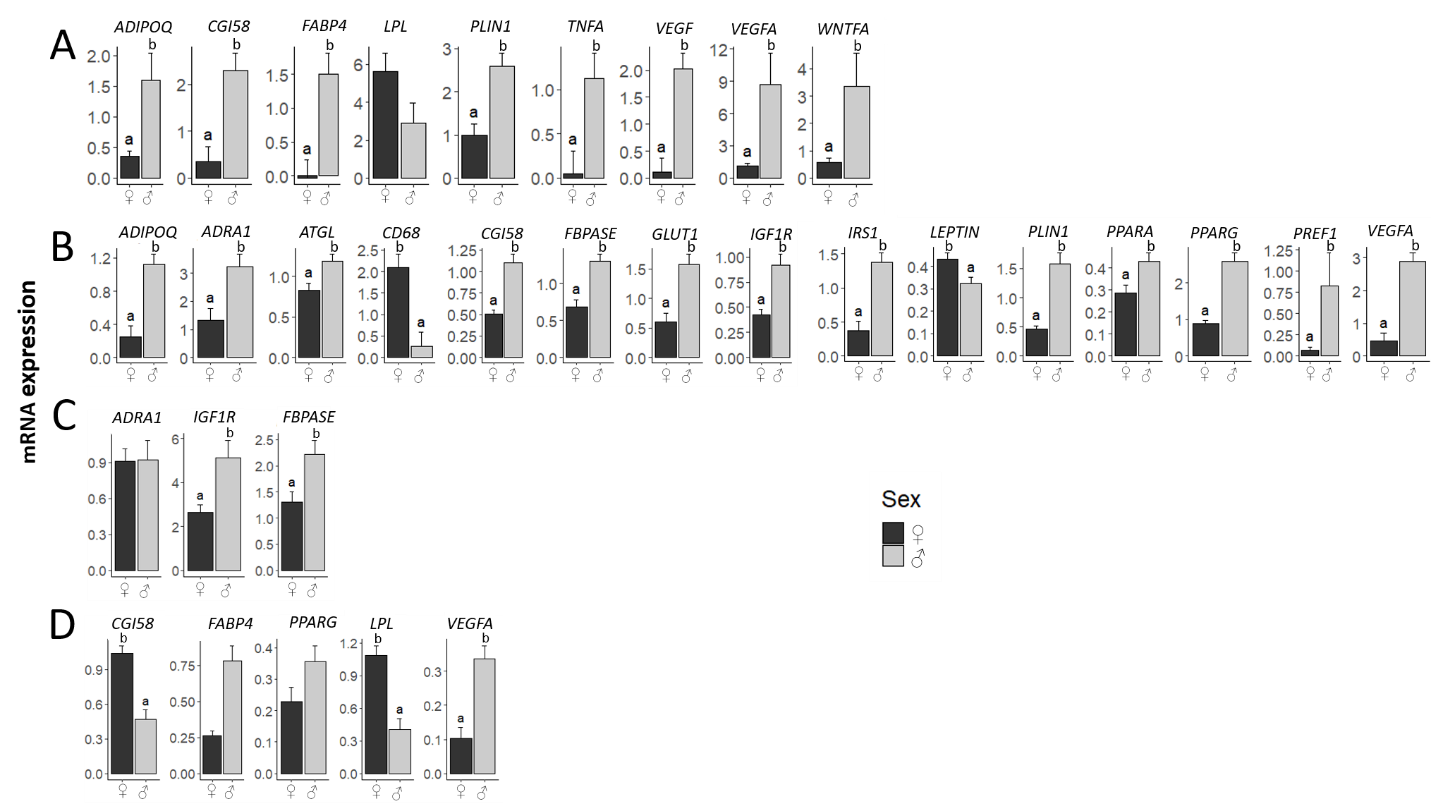
Figure S3** Sex effects, independent of the early nutrition history, on mRNA expression (relative to that of the reference gene, beta-actin) in (A) subcutaneous, (B) perirenal, (C) mesenteric, and (D) epicardial adipose tissues in 2½ years old male (♂) and female (♀) adult sheep.

**
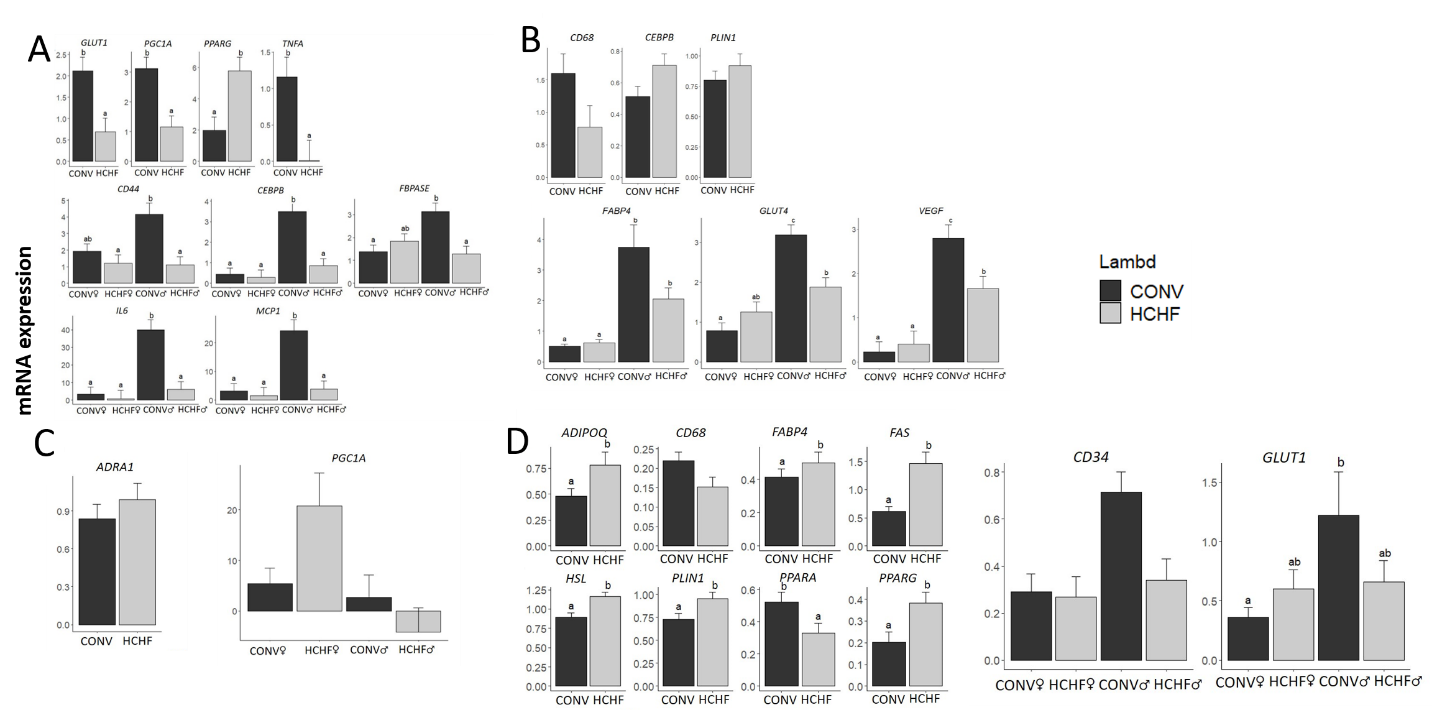
Figure S4** Sex independent effects and sex-dependent interactive effects with early postnatal nutrition on mRNA expressions (relative to that of the reference gene, beta-actin) in (A) subcutaneous, (B) perirenal, (C) mesenteric, and (D) epicardial adipose tissues from 2½ years old male (♂) and female (♀) adult sheep.

**B**

**C**

**D**

**Figure S5** Overview of correlations (r) between different adipocyte size classes with gene expression levels within (A) subcutaneous, (B) perirenal, (C) mesenteric, and (D) epicardial adipose tissues from 2½ year old adult sheep with different early nutrition histories.
