# Supplementary material for: Depot and sex‐specific implications for adipose tissue expandability and functional traits in adulthood of late prenatal and early postnatal malnutrition in a precocial sheep model
Source: Physiol Rep. 2020 Oct 10;8(19):e14600. doi: 10.14814/phy2.14600 (PMC7547587; doi:10.14814/phy2.14600)
Supplement: Supplementary file 2 — Table S1‐S5 [file PHY2-8-e14600-s002.docx]

**Supplementary Tables**

**Table S1** Chemical composition and energy content of experimental feeds

| Feeds | DM (%) | Ash (%) | aNDF (%) | ADF (%) | ADL (%) | CP (%) | Cfat (%) | DE (MJ/kg) |
| --- | --- | --- | --- | --- | --- | --- | --- | --- |
| Prenatal nutrition |  |  |  |  |  |  |  |  |
| Hay | 91.4 | 5.6 | 47.7 | 27 | 3.1 | 20.8 | 4.8 | 13.7 |
| Barley | 89.0 | 2.3 | 14.0 | 6.0 | 1.1 | 12.5 | 3.1 | 17.1 |
| Concentrate | 87.7 | 7.7 | 25.8 | 18 | 2.8 | 15.3 | 3.8 | 12.8 |
| Early postnatal diet | | | | | | | | |
| Hay | 93.1 | 6.8 | 50.4 | 32.3 | 3.5 | 19.1 | 3.7 | 13.5 |
| Maize | 89.5 | 0.6 | 4.1 | <5 | 0.9 | 8.5 | 1.9 | 16.3 |
| Milk powder | 95.6 | 7.1 | - | - | - | 22.5 | 23.6 | 19.2 |
| Cream | 42.9 | 0.8 | - | - | - | 4.3 | 38.0 | 30.5 |

ADF; acid detergent fiber, aNDF; amylase-treated neutral detergent fiber, ADL; acid detergent lignin, CF; crude fat, CP; crude protein, DE; digestible energy, DM; dry matter, and, MJ; metabolizable energy. The feed ingredients used for the experimental diet in the Copenhagen sheep model [5, 18]. The values of the chemical composition and digestible energy of the feed ingredients is based of the percentages of dry matter.

**Table S2** Dietary daily digestible energy and dietary crude protein intake of ewes and lambs

| Parameters | Pre-partum feeding levels (ewes) | | | | | |
| --- | --- | --- | --- | --- | --- | --- |
| Groups | **NORM** | | **HIGH** | | **LOW** | |
| DE (MJ) | 22.9 ± 0.8^b^ | | 34.8 ± 0.6^a^ | | 11.0 ± 0.6^c^ | |
| DCP intake (g) | 263 ± 5.7^b^ | | 284 ± 4.5^a^ | | 125 ± 4.6^c^ | |
|  | **Early postnatal feeding levels (lambs) from day 3 until 6 months of age** | | | | | |
|  | **NORM-CONV** | **NORM-HCHF** | **HIGH-CONV** | **HIGH-HCHF** | **LOW-CONV** | **LOW-HCHF** |
| DE (MJ) |  |  |  |  |  |  |
| Day 3 until 8 weeks | 4.5 ± 0.5^b^ | 8.4 ± 0.5^a^ | 4.3 ± 0.4^b^ | 8.0 ± 0.4^a^ | 4.3 ± 0.4^b^ | 7.7 ± 0.4^a^ |
| 8 weeks until 6 months | 11.9 ± 0.9 | 12.5 ± 0.9 | 12.4 ± 0.7 | 11.5 ± 0.7 | 11.7 ± 0.7 | 10.9 ± 0.7 |
| DCP (g) |  |  |  |  |  |  |
| Day 3 until 8 weeks | 42.9 ± 1.6^a^ | 18.1 ± 1.6^b^ | 41.2 ± 1.3^a^ | 17.2 ± 1.3^b^ | 40.5 ± 1.3^a^ | 17.2 ± 1.3^b^ |
| 8 weeks until 6 months | 122 ± 8.6 ^a^ | 29.8 ± 8.6^b^ | 129 ± 7.1^a^ | 27.2 ± 7.1^b^ | 120 ± 7.1^a^ | 26.7 ± 7.2^b^ |

DCP; dietary crude protein, DE; digestible energy and MJ; metabolizable energy. The daily intake of DE and DCP of ewes and lambs of Copenhagen sheep model [5]. All sheep were born as twins from mothers, which during the last 6 weeks of gestation (term~147 days) had been exposed to NORM (fulfilling 100% of daily energy and protein requirements); HIGH (fulfilling 150% of energy and 110% of protein requirements, respectively); or LOW (50% of NORM) levels of nutrition. From 3-days of age until 6 months of age (post-puberty), one twin was fed a CONV diet (milk replacer during the first 8 weeks of life and exclusively hay thereafter, and adjusted in amounts to achieve moderate constant growth rates of approx. 225 g/day) and the other twin a HCHF diet (high carbohydrate (starch)-high-fat diet (37% fat dairy cream mixed with milk replacer in a 1:1 ratio (max. 2½ l/day) supplemented with rolled maize (max. 2 kg/d)). From 6 months until 2½ years of age, all sheep were fed with the same CONV (low-fat hay-based diet). Tissue samples were obtained at autopsy from the 2½ years old adult sheep, and perirenal and mesenteric adipose tissues stained with Iron-Hematoxylin and EPI with Hematoxylin and Eosin for optimal staining of cell membranes. Values are expressed as means ± SEM with 95% confidence interval. ^abc^ Significant differences between groups are denoted by different superscript letters within row.

**Table S3** Primer sequences and efficiencies

| Gene | Primer sequences | Efficiency |
| --- | --- | --- |
| *ACTB* | F: 5´- ACC CAG ATG ATG TTC GAG ACC TT-3´  R: 5´-TCA CCG GAG TCC ATC ACG AT -3´ | 1.94^[[1]](#footnote-1)^ |
| *ADIPOQ* | F: 5´-ATC AAA CTC TGG AAC CTC CTA TCT AC -3´  R: 5´-TTG CAT TGC AGG CTC AAG -3´ | 1.90^[[2]](#footnote-2)^ |
| *ADRA1* | F: 5´- ATC CAC ACC ATC TCC CTG AG-3´  R: 5´-TCG TCT CTA AGC CCT ACC TCT G -3´ | 2.34^[[3]](#footnote-3)^ |
| *ADRB1* | F: 5´- CGC TCA CCA ACC TCT TCA TC-3´  R: 5´-CAC ACA GGG TCT CAA TGC TG -3´ | 1.943 |
| *ATGL* | F: 5´-CAC CAG CAT CCA GTT CAA CCT -3´  R: 5´-CTG TAG CCC TGT TTG CAC ATC T -3´ | 2.02^[[4]](#footnote-4)^ |
| *CEBPB* | F: 5´-GAC AAG CAC AGC GAC GAG T -3´  R: 5´-GTG CTG CGT CTC CAG GTC -3´ | 3.346^[[5]](#footnote-5)^ |
| *CD34* | F: 5´-TGA CCT GAG AGA GAT GGG CA -3´  R: 5´-CGA GGT GAC CAG TGC AAT CA -3´ | 2.00^[[6]](#footnote-6)^ |
| *CD44* | F: 5´-GAC CAT GGG GCA AAC ACA AC -3´  R: 5´-TCT GCC CAC ACC TTC TCC TA -3´ | 2.036 |
| *CD68* | F: 5´-GTC CTG CTA CCA CCA CCA GT -3´  R: 5´-GCT GGG AAC CAT TAC TCC AA -3´ | 1.92^[[7]](#footnote-7)^ |
| *CGI58* | F: 5´-CAG TGA CGG AAT ACA TCT ACC ACT G -3´  R: 5´-GCC AAT TCG CTG GAG CAT -3´ | 1.99^[[8]](#footnote-8)^ |
| *FABP4* | F: 5´-CAT CTT GCT GAA AGC TCG AC -3´  R: 5´-AGC CAC TTT CCT GGT AGC AA -3´ | 2.16^[[9]](#footnote-9)^ |
| *FAS* | F: 5´-CCC AGC TCA ACG AAA CCA -3´  R: 5´-GAC GAG GTC AAC ACC CTT CC -3´ | 2.08^[[10]](#footnote-10)^ |
| *FBPASE* | F: 5´-CGG GAG ATC AAG TGA AGA AGC T-3´  R: 5´-CAG GTT CGA CTA TGA TGG CAT GT-3´ | 2.02^[[11]](#footnote-11)^ |
| *FTO* | F: 5´-ACA CAT GGC TTC CCT ACC TG -3´  R: 5´-GAG GAT GCG AGA GAC TGG AG -3´ | 2.10^[[12]](#footnote-12)^ |
| *GAPDH* | F: 5´-GTC GGA GTG AAC GGA TTT GG -3´  R: 5´-AAC GAT GTC CAC TTT GCC AGT A -3´ | 2.181 |
| *GcR* | F: 5´-ACT GCC CCA AGT GAA AAC AGA -3´  R: 5´-ATG AAC AGA AAT GGC AGA CAT TTT ATT -3´ | 2.173 |
| *GLUT1* | F: 5´-GCA GGA GAT GAA GGA GGA GAG C -3´  R: 5´-GCA GCA CCA CGG AAA TGA G -3´ | 2.073 |
| *GLUT4* | F: 5´-AGT ATG TGG CGG ATG CTA TGG G -3´  R: 5´-CGG CGG AAG ACG GCT GAG -3´ | 1.943 |
| *HSL* | F: 5´-CCT TCG CAC CAG CCA CAA C-3´  R: 5´-CTC GTC GCC CTC AAA GAA GA-3´ | 2.11^[[13]](#footnote-13)^ |
| *IGF1R* | F: 5´-GCC TTT TAC TCT GTA CCG AAT CG-3´  R: 5´-GCG CTG CAG CCA AGC T-3´ | 1.96^[[14]](#footnote-14)^ |
| *IL6* | F: 5´-TCA TCC TGA GAA GCC TTG AGA -3´  R: 5´-TTT CTG ACC AGA GGA GGG AAT -3 | 1.92^[[15]](#footnote-15)^ |
| *IRSI* | F: 5´-CAA GAC CAT CAG CTT CGT GA -3´  R: 5´-GTC CAC CTG CAT CCA GAA CT -3´ | 1.849 |
| *LEPR* | F: 5´-TTT CCT GGA TGC TGT CAC CC -3´  R: 5´-CGG TTT CCC TAC TCC TTC CG -3´ | 2.01^[[16]](#footnote-16)^ |
| *LEPTIN* | F: 5´-GCT CCA CCC TCT CCT GAG TTT -3´  R: 5´-ACT GGC GAG GAT CTG TTG GT -3´ | 1.9414 |
| *LPL* | F: 5´-CGA GTA TGC AGA AGC TCC AAG TC -3´  R: 5´-CCT GGT GAA CGT GTG TAA AAC ATC -3´ | 1.8414 |
| *MCP1* | F: 5´-GCT GTG ATT TTC AAG ACC ATC CT -3´  R: 5´-GGC CTG CTG GAC CCA TTT -3´ | 1.957 |
| *PGC1A* | F: 5´-CCG AGA ATT CAT GGA GCA AT -3´  R: 5´-GAT TGT GTG TGG GCC TTC TT -3´ | 1.86^[[17]](#footnote-17)^ |
| *PLIN1* | F: 5´-AGG GTG TCA CTG ACA ACG TGG -3´  R: 5´-GTT GTC GAT GTC CCG GAA TT -3´ | 1.94^[[18]](#footnote-18)^ |
| *PPARA* | F: 5´-CGT GTG AAC ATG ACC TAG AAG -3´  R: 5´-ACG AAG GGC GGA TTG TTG -3´ | 1.87^[[19]](#footnote-19)^ |
| *PPARY* | F: 5´-ACG GGA AAG ACG ACA GAC AAA TC -3´  R: 5´-CAC GGA GCG AAA CTG ACA CC -3´ | 2.173 |
| *PREF1* | F: 5´-CCA TGG GCA TCT CTT CCT CA -3´  R: 5´-GCC GGC CTC CTT GGT GAA -3´ | 2.02^[[20]](#footnote-20)^ |
| *TGFB1* | F: 5´-AAG CGG AAG GGC ATC GA -3´  R: 5´-CGA GCC GAA GTT TGG ACA AA -3´ | 2.26^[[21]](#footnote-21)^ |
| *TLR4* | F: 5´-CTG AAT CTC TAC AAA ATC CC -3´  R: 5´-CTT AAT TTC GCA TCT GGA TA -3´ | 2.037 |
| *TNFA* | F: 5´-CCA CCA ACC ATC ACC AAG GA -3´  R: 5´-ACG TTG CGA AGT ATT CCG GT -3´ | 2.12^[[22]](#footnote-22)^ |
| *UCP2* | F: 5´-ATG ACA GAC GAC CTC CCT TG -3´  R: 5´- GGG CAT GAA CCC TTT GTA GA-3´ | 1.90^[[23]](#footnote-23)^ |
| *VEGF* | F: 5´-GGG CTG CTG TAA TGA CGA AAG -3´  R: 5´-TGA GGT TTG ATC CGC ATA ATC TG -3´ | 1.811 |
| *VEGFA* | F: 5´-GCC TTG CCT TGC TGC TCT AC -3´  R: 5´-GGT TTC TGC CCT CCT TCT GC -3´ | 1.987 |
| *WNT5A* | F: 5´-TCT CCT TCG CCC AGG TTG TA -3´  R: 5´-GGC TGT GCT CCT ATG ATA TAT ACT TCT G -3´ | 2.11^[[24]](#footnote-24)^ |

**Table S4** Pre- and early postnatal nutrition interaction effects on adipocyte size distribution in adipose tissue

| Prenatal nutrition | NORM | | HIGH | | LOW | | P-value |
| --- | --- | --- | --- | --- | --- | --- | --- |
| Postnatal nutrition | **CONV** | **HCHF** | **CONV** | **HCHF** | **CONV** | **HCHF** |  |
| BW6 (kg) | 91.13±2.9 | 92.1±3.5 | 98.1±3.0 | 94.7±2.9 | 91.2±2.4 | 99.0±2.7 | 0.12 |
| Cell size classes (µm^2^) and % of adipocytes in each cell size class | | | | | | | |
| SUB | | | | | | | |
| 40-200 | 17.2±1.4 | 11.5±1.5 | 13.3±1.3 | 15.7±1.3 | 13.2±1.1 | 11.7±1.2 | 0.02 |
| PER | | | | | | | |
| 1600-3200 | 19.8±1.4^b^ | 14.4±1.6^ab^ | 11.6±1.4^a^ | 16.5±1.3^ab^ | 17.5±1.2^b^ | 17.2±1.2^ab^ | 0.02 |
| 6400-12800 | 12.5±2.3^a^ | 22.4±2.7^ab^ | 25.0±2.3^b^ | 18.7±2.2^ab^ | 14.3±2.1^a^ | 18.1±2.1^ab^ | 0.01 |
| 12800-25600 | 3.00±0.8^a^ | 6.74±2.3^ab^ | 10.2±2.6^b^ | 3.03±0.8^a^ | 8.04±2.3^a^ | 8.37±2.6^ab^ | 0.005 |

Only parameters for which significant effects were obtained are shown. All sheep were born as twins from mothers, which during the last 6 weeks of gestation (term~147 days) had been exposed to NORM (fulfilling 100% of daily energy and protein requirements); HIGH (fulfilling 150% of energy and 110% of protein requirements, respectively); or LOW (50% of NORM) levels of nutrition. From 3-days of age until 6 months of age (post-puberty), one twin was fed a CONV diet (milk replacer during the first 8 weeks of life and exclusively hay thereafter, and adjusted in amounts to achieve moderate constant growth rates of approx. 225 g/day) and the other twin a HCHF diet (high carbohydrate (starch)-high-fat diet (37% fat dairy cream mixed with milk replacer in a 1:1 ratio (max. 2½ l/day) supplemented with rolled maize (max. 2 kg/d)). From 6 months until 2½ years of age, all sheep were fed with the same CONV (low-fat hay-based diet). Tissue slides were obtained at autopsy from the 2½ years old adult sheep, and adipose tissues stained with Iron-Hematoxylin. Average cross-sectional area (CSA) of adipocytes and distribution of adipocytes in cell size classes were determined in whole tissue scans using the Iron Haematoxylin Adipose Tissue software (APP ID 10113; Visiopharm®, Hoersholm, Denmark). Values are expressed as emmean ± SEM derived from proportions (%) with 95% confidence interval. The number of animals in the pre- and postnatal nutrition groups are: Subcutaneous adipose tissue: NORM-CONV (N=5); NORM-HCHF (N=4); HIGH-CONV (N=6); HIGH-HCHF (N=6); LOW-CONV (N=8) and; LOW-HCHF (N=7); Perirenal adipose tissue: NORM-CONV (N=6); NORM-HCHF (N=4); HIGH-CONV (N=6); HIGH-HCHF (N=6); LOW-CONV (N=8) and; LOW-HCHF (N=7).

**Table S5** Effects of early postnatal nutrition on adipocyte size and size distribution in sheep.

|  | Postnatal nutrition | | | | P-value |
| --- | --- | --- | --- | --- | --- |
|  | **CONV** | | **HCHF** | |  |
| Sex: | ♂ | ♀ | ♂ | ♀ |  |
| Average CSA (µm^2^) | | | | | |
| PER | 4632±233 | | 4541±259 | | 0.05 |
| EPI | 2099±72^a^ | | 2355±78^b^ | | 0.03 |
| Cell size class (µm^2^) and % of adipocytes in each cell size class | | | | | |
| PER | | | | | |
| 3200-6400 | 18.4±1.1^a^ | | 21.9±1.2^b^ | | 0.03 |
| 25600-36000 | 0.19±0.04^a^ | 0.87±0.13^c^ | 0.25±0.05^ab^ | 0.46±0.09^bc^ | 0.01 |
| MES | | | | | |
| 6400-12800 | 9.12±1.63^a^ | 15.99±1.30^b^ | 15.89±1.69^b^ | 15.33±1.78^ab^ | 0.02 |
| EPI | | | | | |
| 40-200 | 5.19±0.26 | | 5.90±0.33 | | 0.01 |
| 800-1600 | 17.00±0.60^a^ | 20.00±0.58^b^ | 18.00±0.61^ab^ | 17.60±0.63^a^ | 0.01 |
| 1600-3200 | 30.20±1.26^a^ | 35.10±1.20^b^ | 26.50±1.05^a^ | 26.30±1.11^a^ | 0.05 |
| 3200-6400 | 24.50±1.65^b^ | 15.20±1.45^a^ | 22.00±1.72^b^ | 20.20±1.66^ab^ | 0.02 |
| 6400-12800 | 1.87 ± 0.29^b^ | 0.86 ± 0.11^a^ | 3.98 ± 0.59^c^ | 4.71±0.74^c^ | 0.004 |
| 12800-25600 | 0.15 ± 0.02^a^ | 0.11± 0.01^a^ | 0.31 ± 0.04^b^ | 0.43 ± 0.07^b^ | 0.02 |
| 25600-36000 | 0.03±0.00^a^ | | 0.05±0.01^b^ | | 0.003 |

Only parameters for which significant effects were obtained are shown. All sheep were born as twins from mothers, which during the last 6 weeks of gestation (term~147 days) had been exposed to NORM (fulfilling 100% of daily energy and protein requirements); HIGH (fulfilling 150% of energy and 110% of protein requirements, respectively); or LOW (50% of NORM) levels of nutrition. From 3-days of age until 6 months of age (post-puberty), one twin was fed a CONV diet (milk replacer during the first 8 weeks of life and exclusively hay thereafter, and adjusted in amounts to achieve moderate constant growth rates of approx. 225 g/day) and the other twin a HCHF diet (high carbohydrate (starch)-high-fat diet (37% fat dairy cream mixed with milk replacer in a 1:1 ratio (max. 2½ l/day) supplemented with rolled maize (max. 2 kg/d)). From 6 months until 2½ years of age, all sheep were fed with the same CONV (low-fat hay-based diet). Tissue samples were obtained at autopsy from the 2½ years old adult sheep, and perirenal and mesenteric adipose tissues stained with Iron-Hematoxylin and EPI with Hematoxylin and Eosin for optimal staining of cell membranes. Average cross-sectional area (CSA) of adipocytes and distribution of adipocytes in cell size classes were determined in whole tissue scans using the Iron Haematoxylin Adipose Tissue software (APP ID 10113; Visiopharm®, Hoersholm, Denmark). Values are expressed as emmean ± SEM derived from proportions (%) with 95% confidence interval. Number of animals in the different groups: CONV (N=20, 8♂:12♀) and HCHF (N=17, 9♂:8♀).

1. Safayi S, Theil PK, Hou L, Engbæk M, Nørgaard JV, Sejrsen K, et al. Continuous lactation effects on mammary remodeling during late gestation and lactation in dairy goats. Journal of Dairy Science. 2010;93(1):203-17. [↑](#footnote-ref-1)
2. Muhlhausler BS, Duffield J, McMillen I. Increased maternal nutrition stimulates peroxisome proliferator activated receptor-γ, adiponectin, and leptin messenger ribonucleic acid expression in adipose tissue before birth. Endocrinology. 2007;148(2):878-85. [↑](#footnote-ref-2)
3. Chan LL, Sebert SP, Hyatt MA, Stephenson T, Budge H, Symonds ME, et al. Effect of maternal nutrient restriction from early-to-mid gestation on cardiac function and metabolism after adolescent-onset obesity. American Journal of Physiology-Regulatory, Integrative and Comparative Physiology. 2009. [↑](#footnote-ref-3)
4. Ji P, Osorio J, Drackley J, Loor J. Overfeeding a moderate energy diet prepartum does not impair bovine subcutaneous adipose tissue insulin signal transduction and induces marked changes in peripartal gene network expression. Journal of Dairy Science. 2012;95(8):4333-51 [↑](#footnote-ref-4)
5. González-Calvo L, Joy M, Blanco M, Dervishi E, Molino F, Sarto P, et al. Effect of vitamin E supplementation or alfalfa grazing on fatty acid composition and expression of genes related to lipid metabolism in lambs. Journal of Animal Science. 2015;93(6):3044-54. [↑](#footnote-ref-5)
6. Ceccarelli G, Pozzo E, Scorletti F, Benedetti L, Cusella G, Ronzoni FL, et al. Molecular signature of amniotic fluid derived stem cells in the fetal sheep model of myelomeningocele. Journal of Pediatric Surgery. 2015;50(9):1521-7. [↑](#footnote-ref-6)
7. Khanal P, Pandey D, Binti Ahmad S, Safayi S, Kadarmideen HN, Olaf Nielsen M. Differential impacts of late gestational over–and undernutrition on adipose tissue traits and associated visceral obesity risk upon exposure to a postnatal high‐fat diet in adolescent sheep. Physiological Reports. 2020;8(3):e14359. [↑](#footnote-ref-7)
8. Ahn J, Li X, Choi YM, Shin S, Oh S-A, Suh Y, et al. Differential expressions of G0/G1 switch gene 2 and comparative gene identification-58 are associated with fat content in bovine muscle. Lipids. 2014;49(1):1-14. [↑](#footnote-ref-8)
9. Hosseini A, Behrendt C, Regenhard P, Sauerwein H, Mielenz M. Differential effects of propionate or β‐hydroxybutyrate on genes related to energy balance and insulin sensitivity in bovine white adipose tissue explants from a subcutaneous and a visceral depot 1. Journal of Animal Physiology and Animal Nutrition. 2012;96(4):570-80. [↑](#footnote-ref-9)
10. Qiao Y, Huang Z, Li Q, Liu Z, Hao C, Shi G, et al. Developmental changes of the FAS and HSL mRNA expression and their effects on the content of intramuscular fat in Kazak and Xinjiang sheep. Journal of Genetics and Genomics. 2007;34(10):909-17. [↑](#footnote-ref-10)
11. Van Harten S, Brito R, Almeida A, Scanlon T, Kilminster T, Milton J, et al. Gene expression of regulatory enzymes involved in the intermediate metabolism of sheep subjected to feed restriction. Animal. 2013;7(3):439-45. [↑](#footnote-ref-11)
12. Siersbæk R, Nielsen R, Mandrup S. PPARγ in adipocyte differentiation and metabolism–Novel insights from genome‐wide studies. FEBS letters. 2010;584(15):3242-9. [↑](#footnote-ref-12)
13. Xu X, Wei X, Yang Y, Niu W, Kou Q, Wang X, et al. PPARγ, FAS, HSL mRNA and protein expression during Tan sheep fat-tail development. Electronic Journal of Biotechnology. 2015;18(2):122-7. [↑](#footnote-ref-13)
14. Wallace JM, Milne JS, Aitken RP, Adam CL. Influence of birth weight and gender on lipid status and adipose tissue gene expression in lambs. Journal of Molecular Endocrinology. 2014;53(1):131-44. [↑](#footnote-ref-14)
15. Wang H, Zhao J, Huang Y, Yan X, Meyer A, Du M, et al. Effects of maternal plane of nutrition and increased dietary selenium in first-parity ewes on inflammatory response in the ovine neonatal gut. Journal of Animal Science. 2012;90(1):325-33. [↑](#footnote-ref-15)
16. Accession no. NM_001009763.1 [↑](#footnote-ref-16)
17. Struewing IT, Barnett CD, Tang T, Mao CD. Lithium increases PGC‐1α expression and mitochondrial biogenesis in primary bovine aortic endothelial cells. The FEBS journal. 2007;274(11):2749-65. [↑](#footnote-ref-17)
18. Sanz A, Serrano C, Ranera B, Dervishi E, Zaragoza P, Calvo J, et al. Novel polymorphisms in the 5′ UTR of FASN, GPAM, MC4R and PLIN1 ovine candidate genes: Relationship with gene expression and diet. Small Ruminant Research. 2015;123(1):70-4. [↑](#footnote-ref-18)
19. Bispham J, Gardner D, Gnanalingham M, Stephenson T, Symonds M, Budge H. Maternal nutritional programming of fetal adipose tissue development: differential effects on messenger ribonucleic acid abundance for uncoupling proteins and peroxisome proliferator-activated and prolactin receptors. Endocrinology. 2005;146(9):3943-9. [↑](#footnote-ref-19)
20. Fahrenkrug SC, Freking BA, Smith TP. Genomic organization and genetic mapping of the bovine PREF-1 gene. Biochemical and Biophysical Research Communications. 1999;264(3):662-7 [↑](#footnote-ref-20)
21. Hacariz O, Sayers G, Flynn R, Lejeune A, Mulcahy G. IL‐10 and TGF‐β1 are associated with variations in fluke burdens following experimental fasciolosis in sheep. Parasite Immunology. 2009;31(10):613-22. [↑](#footnote-ref-21)
22. Johnsen L, Kongsted AH, Nielsen MO. Prenatal undernutrition and postnatal overnutrition alter thyroid hormone axis function in sheep. Journal of Endocrinology. 2013;216(3):389-402. [↑](#footnote-ref-22)
23. Yiallourides M, Sebert S, Wilson V, Sharkey D, Rhind S, Symonds M, et al. The differential effects of the timing of maternal nutrient restriction in the ovine placenta on glucocorticoid sensitivity, uncoupling protein 2, peroxisome proliferator-activated receptor-γ and cell proliferation. Reproduction. 2009;138(3):601-8. [↑](#footnote-ref-23)
24. Kiewisz J, Kaczmarek MM, Morawska E, Blitek A, Kapelanski W, Ziecik AJ. Estrus synchronization affects WNT signaling in the porcine reproductive tract and embryos. Theriogenology. 2011;76(9):1684-94 [↑](#footnote-ref-24)
